# Vegetable intake and the risk of bladder cancer in the BLadder Cancer Epidemiology and Nutritional Determinants (BLEND) international study

**DOI:** 10.1186/s12916-021-01931-8

**Published:** 2021-03-09

**Authors:** Evan Yi-Wen Yu, Anke Wesselius, Siamak Mehrkanoon, Mieke Goosens, Maree Brinkman, Piet van den Brandt, Eric J. Grant, Emily White, Elisabete Weiderpass, Florence Le Calvez-Kelm, Marc J. Gunter, Inge Huybrechts, Elio Riboli, Anne Tjonneland, Giovanna Masala, Graham G. Giles, Roger L. Milne, Maurice P. Zeegers

**Affiliations:** 1 Department of Complex Genetics and Epidemiology, School of Nutrition and Translational Research in Metabolism, Maastricht University, Universiteitssingel 40 (Room C5.570), 6229 ER Maastricht, the Netherlands; 2 CAPHRI School for Public Health and Primary Care, Maastricht University, Maastricht, The Netherlands; 3 Department of Data Science and Knowledge Engineering, Maastricht University, Maastricht, The Netherlands; 4 Department of General Practice, Katholieke Universiteit Leuven, ACHG-KU Leuven, Leuven, Belgium; 5 Department of Clinical Studies and Nutritional Epidemiology, Nutrition Biomed Research Institute, Melbourne, Australia; 6 Cancer Epidemiology Division, Cancer Council Victoria, 615 St Kilda Road, Melbourne, Victoria 3004 Australia; 7 Department of Epidemiology, Schools for Oncology and Developmental Biology and Public Health and Primary Care, Maastricht University Medical Centre, Maastricht, The Netherlands; 8 Department of Epidemiology Radiation Effects Research Foundation, Hiroshima, Japan; 9 Fred Hutchinson Cancer Research Center, Seattle, WA USA; 10 International Agency for Research on Cancer World Health Organization, Lyon, France; 11 Department of Epidemiology and Biostatistics, School of Public Health, Imperial College London, London, UK; 12 Danish Cancer Society Research Center, Copenhagen, Denmark; 13 Department of Public Health, University of Copenhagen, Copenhagen, Denmark; 14 Molecular and Lifestyle Epidemiology Branch, Cancer Risk Factors and Lifestyle Epidemiology Unit, Institute for Cancer Research, Prevention and Clinical Network ISPRO, Florence, Italy; 15 Centre for Epidemiology and Biostatistics, Melbourne School of Population and Global Health, The University of Melbourne, 207 Bouverie Street, Melbourne, Victoria 3010 Australia; 16 Precision Medicine, School of Clinical Sciences at Monash Health, Monash University, Clayton, Victoria 3168 Australia; 17 School of Cancer Sciences, University of Birmingham, Birmingham, UK

**Keywords:** Bladder cancer, Vegetable, Dietary diversity analysis, Cohort study

## Abstract

**Background:**

Although a potential inverse association between vegetable intake and bladder cancer risk has been reported, epidemiological evidence is inconsistent. This research aimed to elucidate the association between vegetable intake and bladder cancer risk by conducting a pooled analysis of data from prospective cohort studies.

**Methods:**

Vegetable intake in relation to bladder cancer risk was examined by pooling individual-level data from 13 cohort studies, comprising 3203 cases among a total of 555,685 participants. Pooled multivariate hazard ratios (HRs), with corresponding 95% confidence intervals (CIs), were estimated using Cox proportional hazards regression models stratified by cohort for intakes of total vegetable, vegetable subtypes (i.e. non-starchy, starchy, green leafy and cruciferous vegetables) and individual vegetable types. In addition, a diet diversity score was used to assess the association of the varied types of vegetable intake on bladder cancer risk.

**Results:**

The association between vegetable intake and bladder cancer risk differed by sex (*P*-interaction = 0.011) and smoking status (*P*-interaction = 0.038); therefore, analyses were stratified by sex and smoking status. With adjustment of age, sex, smoking, energy intake, ethnicity and other potential dietary factors, we found that higher intake of total and non-starchy vegetables were inversely associated with the risk of bladder cancer among women (comparing the highest with lowest intake tertile: HR = 0.79, 95% CI = 0.64–0.98, *P* = 0.037 for trend, HR per 1 SD increment = 0.89, 95% CI = 0.81–0.99; HR = 0.78, 95% CI = 0.63–0.97, *P* = 0.034 for trend, HR per 1 SD increment = 0.88, 95% CI = 0.79–0.98, respectively). However, no evidence of association was observed among men, and the intake of vegetable was not found to be associated with bladder cancer when stratified by smoking status. Moreover, we found no evidence of association for diet diversity with bladder cancer risk.

**Conclusion:**

Higher intakes of total and non-starchy vegetable are associated with reduced risk of bladder cancer for women. Further studies are needed to clarify whether these results reflect causal processes and potential underlying mechanisms.

**Supplementary Information:**

The online version contains supplementary material available at 10.1186/s12916-021-01931-8.

## Background

Bladder cancer is the most common malignancy of urinary tract, with an estimated 550,000 new cases and 200,000 deaths annually [1–3]. Incidence rates of bladder cancer are highest in Europe and North America, with a strong predominance in men and the elderly [4–9]. Due to its high rate of recurrence, bladder cancer places costly burden on healthcare systems in terms of life time treatment [10]. Apart from well-established risk factors, such as smoking, occupational exposure and arsenic contamination [11–15], dietary factors may also contribute to bladder cancer development considering that many dietary compounds are excreted in urine and thereby come into direct contact with the bladder epithelium [12]. As recommended in numerous dietary guidelines globally, intake of vegetables may be beneficial for cancer prevention due to their rich content of vitamins, fibre, minerals, and other bioactive compounds [16–18]. However, the World Cancer Research Fund (WCRF) International’s Continuous Update Project (CUP) 2018 [19] found that the evidence suggesting that higher intake of vegetable decreases the risk of bladder cancer is limited.

Previous epidemiologic studies on the influence of vegetable intake on bladder cancer risk have yielded inconsistent results. While most studies reported null association between total vegetable intake and bladder cancer risk, some showed conflicting results, with some showing inverse associations [20–24], and others that a higher intake increases the risk of bladder cancer [25]. A meta-analysis based on cohort studies only found no association between vegetable intake and bladder cancer risk [26], while those combining results from case-control and cohort studies showed an inverse association [27–29].

It remains unclear which vegetable subtype (e.g. non-starchy, green leafy or cruciferous) or individual vegetable type (e.g. broccoli, garlic, cauliflower, kale, cabbage, asparagus) may be responsible for a possible inverse association with bladder cancer. The potential protective effect could, therefore, be the result of a cumulative influence of many preventative agents from total vegetable intake, or from a anticarcinogenic property of agents in a specific vegetable (i.e. isothiocyanates from broccoli [30–32], S-allylmercaptocysteine (SAMC) [33], and diallyl trisulfide (DATS) [34] from garlic).

This inconsistent evidence and lack of knowledge on which vegetable or which subtypes are possibly associated with bladder cancer risk could be due to the small sample sizes of previous studies and their consequent lack of statistical power to detect weak associations and perform subgroup analyses [27]. Therefore, this study aimed to obtain more conclusive results on the associations between intake of total vegetable and vegetable subtypes and bladder cancer risk by pooling data from 555,685 participants with 3203 bladder cancer cases in 13 cohort studies included in the BLadder Cancer Epidemiology and Nutritional Determinants (BLEND) international consortium.

## Methods

### Study sample

Data were obtained from BLEND, an international nutritional consortium currently consisting of 19 case-control studies and 16 cohort studies. Thirteen cohort studies with a total of 555,685 participants, 3203 of whom developed incident bladder cancer, had sufficient information on vegetable intake to be eligible for inclusion in the present study. These studies originated from 12 countries in 4 continents [i.e. Europe: European Prospective Investigation into Cancer and Nutrition cohort study (EPIC) [35] (Denmark [36], France [37], Germany [38], Italy [39], Spain [40], Sweden [41, 42], The Netherlands [43], UK [44, 45], Norway [46]), NetherLands Cohort Study (NLCS) [47]; North America: VITamins And Lifestyle cohort study (VITAL) [48]; Oceania: Melbourne Collaborative Cohort Study (MCCS) in Australia [49, 50]; and Asia: Radiation Effects Research Foundation-Life Span Study (RERF-LSS) [51]]. Person-years of follow-up for each participant were calculated from the date of study enrolment until the date of bladder cancer diagnosis, or date of last follow-up (e.g. date of death, lost to follow-up, or study exit), whichever came first. For the NLCS study, a nested case-cohort design was applied in order to increase the follow-up coverage and efficiency, in which the number of person-years at risk was estimated based on a sub-cohort that was randomly sampled [47]. Each study was approved by their local ethical research committee [35, 47, 48, 50, 51] (Additional file 1: Table S1).

### Data collection and coding

Details on the methodology of the BLEND consortium have been described elsewhere [52]. In brief, all included studies used self-administered or trained interviewer administered food frequency questionnaire (FFQ) that was validated on either food groups [48, 53–56], and/or energy intake [53, 56, 57]. For each study, participants were asked to report on their usual vegetable intake during the year before study enrolment. This data was harmonised and categorised in individual vegetable subtype intake using the hierarchal Eurocode 2 food coding system developed by the European Union [58], with weekly, monthly or yearly intake converted to grams (g) per day. The classification of the Eurocode 2 Food Coding System defines which food items are covered for what food category so that the potential for misidentification of foods will be limited.

Each study ascertained incident bladder cancer, defined to include all urinary bladder neoplasms according to the International Classification of Diseases for Oncology (ICD-O-3 code C67) using population-based cancer registries, health insurance records, or medical records. Bladder cancer was classified as non-muscle invasive bladder cancer (NMIBC) or muscle invasive bladder cancer (MIBC). NMIBC included non-invasive papillary carcinomas confined to the urothelium (stage Ta), carcinomas that invaded the lamina propria of the bladder wall (stage T1) and high grade flat non-invasive carcinomas confined to the urothelium (carcinoma in situ; CIS). MIBC included carcinomas that invaded into the detrusor muscle (stage T2), carcinomas that invaded into the peri-vesical tissue (stage T3) and carcinomas that invaded adjacent tissues and organs (most often the prostate or uterus, stage T4).

In addition to information on vegetable and other dietary intakes, the BLEND dataset also included data on study characteristics (design, method of dietary assessment, geographical region), participant demographics (age, sex and ethnicity), smoking status and smoking pack-years (i.e. the number of cigarettes smoked per day multiplying the years of smoking), which were measured at baseline.

### Statistical analyses

To assess the influence of vegetable intake on bladder cancer risk, Cox regression analyses with a stratification approach to adjust for cross-cohort heterogeneity [59] were used to estimate the pooled hazard ratios (HRs) and 95% confidence intervals (CIs). The proportional hazard assumption was examined for each analysis and no evidence of violation was found. Additionally, the appropriateness of the use of the log-normal distribution was tested using a Wald test, and again, no evidence of violation was found. The intake of total vegetable and the main vegetable subgroups [i.e. non-starchy (including 3162 cases/545,951), starchy (including 1514 cases/96,108 participants), green leafy (including 3162 cases/574,381 participants) and cruciferous (including 3162/574,381 participants) (Additional file 1: Table S2)] were expressed as tertiles: low intake (tertile 1), medium intake (tertile 2) and high intake (tertile 3). Low intake was used as the reference group and associations were assessed applying two models. Model 1 was adjusted for age (years), sex (men and women), smoking and total energy intake [kilocalorie (kcal)/day, continuous]. Model 2 was additionally adjusted for ethnicity (Caucasian or non-Caucasian), alcohol intake [millilitre (ml)/day, continuous], sugar intake [gram (g)/day, continuous], meat intake (g/day, continuous), grain intake (g/day, continuous) and fat intake (g/day, continuous), which have been shown to be potential dietary risk factors for bladder cancer risk [60]. Smoking was defined as a dummy variable: 0 (never smokers); 1 [current light smokers (i.e. smoking less than 20 pack-years)]; 2 [current heavy smokers (i.e. smoking more than 20 pack-years)]; 3 [current smokers (no information on pack-years)]; 4 [former light smokers (i.e. smokers who ceased smoking over 1 year prior and smoked less than 20 pack-years)]; 5 [former heavy smokers (i.e. smokers who ceased smoking over 1 year prior and smoked more than 20 pack-years)]; and 6 [former smokers (smokers who ceased smoking over 1 year prior and no information on pack-years)]. Total energy intake was included in the models in addition to the energy adjustment of the residual model [61]. To understand the relevance of effect modification, the main interaction terms between tertiles of vegetable intake and age, sex and smoking status were added to model 1. *P*-interaction < 0.05 was considered statistically significant where upon all analyses were stratified for the covariate of interest. A supplementary analysis was performed to examine the association of individual vegetables with bladder cancer risk based on the eligible cohorts with sufficient data.

In addition, the HRs and 95% CIs of bladder cancer per 1 standard deviation (SD) increase in total vegetable and vegetable subgroups intake were estimated using the same models. To test for linearity or nonlinearity, we included both linear and quadratic terms (i.e. the absolute intake and intake squared) in the models and then used a likelihood ratio test to assess the difference between the nonlinear and linear models [62]. Since results showed no evidence of a non-linear association, linear models were applied. P for trend test was conducted by assigning medians to per 1 SD increase in intake as a continuous variable in the models.

The variables of bladder cancer status (i.e. cases or non-cases), follow-up time, age, sex, smoking and total energy intake were complete without missing values. Missing values of variables (e.g. categorical: ethnicity; continuous: alcohol intake, sugar intake, meat intake, vegetable intake, and fat intake; missing proportion were all less than 5%) were imputed by multiple imputation method. Since study protocols and characteristics of participants across cohorts might differ, multiple imputation was done separately for each included cohort study. Only participants with complete information on bladder cancer status, age, sex, smoking and total energy intake were included in building of the imputation models. Linear regression models were then fitted for those variables with missing data separately.

Besides assessing of the vegetable quantity, also the variety of vegetables intake was assessed. For this, a diet diversity score (DDS) was used to quantify the variety of vegetable intake [52, 53] and to assess the association of the varied types of vegetable intake on bladder cancer risk. DDS was calculated as the total number of different vegetable types reported according to the Eurocode 2 food coding system (i.e. leafy vegetables, Brassicas, stalk vegetables, shoot vegetables, onion-family vegetables, tubers, root vegetables, fruit vegetables, pod and seed vegetables, edible fungi and seaweeds) consumed at least once per week, without taking into account a minimum intake, and was then divided in 4 groups: 0–2 low diversity, 2–4 median diversity, 4–6 high diversity and 6–8 very high diversity. To analyse the effect of variety in vegetable intake independent from the amount of vegetable intake, we adjusted the total vegetable intake for the DDS assessment.

A sensitivity analysis was performed by removing cases diagnosed within the first 2 years after recruitment to each study. Stratified analyses were performed for total and non-starchy vegetables by both sex and smoking status. In addition, the association between intake of total vegetable and risk of bladder cancer was assessed in each participating cohort separately and combined in a meta-analysis approach using a random-effect model. Finally, multiple testing for individual vegetable intake was performed by using false discovery rate (FDR) method.

All statistical analyses were performed using STATA version 14 SE (Stata Corporation, TX, USA) and R 4.0.2.. A two-tailed *P* values < 0.05 was considered statistically significant.

## Results

Baseline characteristics of the included study samples are presented in Table 1. In total, 555,685 study participants contributed 6,276,560 person-years of follow-up over a median of 11 years, with 3203 incident bladder cancer cases (2405 men, 798 women) diagnosed. Of these, 2040 (64%) cases had available diagnosis records of NMIBC (40%) or MIBC (24%). The median age at baseline was 60 years for cases and 53 years for non-cases. The majority (98.2%) of participants were Caucasian. Higher intake of total vegetable, non-starchy vegetables, green leafy vegetable and cruciferous vegetable were observed in bladder cancer cases (*P* value < 0.001, < 0.001, 0.476 and < 0.001 respectively), while only higher intake of starchy vegetables was observed in non-bladder-cancer cases (*P* value < 0.001).

**Table 1:** Characteristics of the study population (3203 cases and 552,482 non-cases) and vegetable intake

Characteristics		Vegetable intake (g/day)					***P*** value^**a**^	***P***-interaction
		Total	Low (0–117)	Medium (117–221)	High (> 221)	Mean [g/day (SD)]		
***N*** **(%)**		555,685	185,229 (33.33)	185,228 (33.33)	185,228 (33.33)	197.50 (146.00)	< 0.001	
Case (%)		3203	962 (30.03)	916 (28.60)	1325 (41.37)	207.37 (143.69)		
NMIBC (%)		1267	392 (30.94)	390 (30.78)	485 (38.28)	202.11 (131.77)		
MIBC (%)		773	167 (21.60)	213 (27.55)	393 (50.84)	238.49 (138.60)		
Non-case (%)		552,482	184,267 (33.35)	184,312 (33.36)	183,903 (33.29)	197.44 (146.01)		
**Person-years**		6,276,560	2,014,670	2,054,381	2,207,509	197.50 (146.00)	< 0.001	
**Sex**								0.011
Male (%)	Case	2405	717 (29.81)	646 (26.86)	1042 (43.33)	212.72 (148.17)	< 0.001	
	Non-case	178,330	78,331 (43.92)	56,376 (31.61)	43,623 (24.46)	175.30 (147.18)		
Female (%)	Case	798	245 (30.70)	270 (33.83)	283 (35.46)	191.17 (127.91)	0.089	
	Non-case	374,152	105,936 (28.31)	127,936 (34.19)	140,280 (37.49)	208.19 (144.22)		
**Age [mean (SD)]** ^**b**^	Case	3203	60.01 (8.42)	60.01 (7.39)	60.86 (6.86)	207.37 (143.69)	< 0.001	0.436
	Non-case	552,482	53.36 (10.40)	52.31 (9.90)	51.95 (10.35)	197.44 (146.01)		
**Smoking**								0.038
**Smoking status**								
Current (%)	Case	1201	380 (31.64)	339 (28.23)	482 (40.13)	201.13 (135.44)	< 0.001	
	Non-case	113,490	44,430 (39.15)	39,551 (34.85)	29,509 (26.00)	183.18 (146.82)		
Former (%)	Case	1353	391 (28.90)	390 (28.82)	572 (42.28)	213.87 (153.47)	< 0.001	
	Non-case	163,897	56,559 (34.51)	54,683 (33.36)	52,655 (32.13)	190.83 (142.55)		
Never (%)	Case	649	191 (29.43)	187 (28.81)	271 (41.76)	205.48 (137.14)	0.476	
	Non-case	275,095	83,278 (30.27)	90,078 (32.74)	101,739 (36.98)	207.22 (146.95)		
**Smoking pack-years [mean (SD)]** ^**c**^		147,303	25.01 (19.92)	22.44 (17.69)	22.38 (19.21)	194.75 (145.85)	< 0.001	
**Ethnicity (%)**		555,406	185,043 (33.32)	185,155 (33.34)	185,208 (33.35)	197.54 (146.00)		
Caucasian		545,114	175,717 (32.23)	184,388 (33.83)	185,009 (33.94)	200.22 (145.76)	< 0.001	
Non-Caucasian		10,292	9326 (90.61)	767 (7.45)	199 (1.93)	49.29 (53.49)	0.590	
**Total energy intake [kcal/day (SD)]**		555,685	2229.87 (1939.18)	2071.28 (670.85)	2217.35 (713.94)	197.50 (146.00)	< 0.001	
**Dietary diversity score [mean (SD)]**		555,685	6.69 (2.04)	6.82 (2.39)	6.71 (2.57)	197.50 (146.00)	< 0.001	
**Alcohol intake [g/day (SD)]**		555,685	4.28 (10.20)	3.19 (8.34)	2.20 (6.43)	197.50 (146.00)	< 0.001	
**Sugar intake [g/day (SD)]**		555,685	23.20 (39.85)	23.55 (46.67)	27.55 (64.98)	197.50 (146.00)	< 0.001	
**Meat intake [g/day (SD)]**		555,685	70.63 (81.39)	76.63 (53.73)	85.65 (61.73)	197.50 (146.00)	< 0.001	
**Grain intake [g/day (SD)]**		555,685	289.53 (295.21)	294.85 (177.10)	296.94 (174.82)	197.50 (146.00)	0.003	
**Fruit intake [g/day (SD)]**		555,685	80.38 (85.44)	118.59 (102.57)	168.48 (131.14)	197.50 (146.00)	0.268	
**Fat intake [g/day (SD)]**		555,685	25.45 (26.45)	23.64 (16.10)	28.37 (20.28)	197.50 (146.00)	< 0.001	
		**Non-starchy vegetable intake**						
		Total	Low (0–108)	Medium (108–211)	High (> 211)	Mean [g/day (SD)]		
		549,113	183,038 (33.33)	183,114 (33.35)	182,961 (33.32)	194.52 (146.02)	< 0.001	
Case (%)		3162	821 (25.96)	771 (24.38)	1570 (49.65)	201.44 (141.33)		
Non-case (%)		545,951	182,217 (33.38)	182,343 (33.40)	181,391 (33.22)	194.49 (146.04)		
		**Starchy vegetable intake**						
		Total	Low (0–7)	Medium (7–18)	High (> 18)	Mean [g/day (SD)]		
		97,622	32,698 (33.33)	32,698 (33.49)	32,383 (33.17)	17.69 (20.15)	< 0.001	
Case (%)		1514	692 (45.71)	491 (32.43)	331 (21.86)	12.74 (12.74)		
Non-case (%)		96,108	31,849 (33.14)	32,207 (33.51)	32,052 (33.35)	17.77 (20.20)		
		**Green leafy vegetable intake**						
		Total	Low (0–5)	Medium (5–23)	High (> 23)	Mean [g/day (SD)]		
		549,113	183,039 (33.33)	183,037 (33.33)	183,037 (33.33)	23.85 (35.29)	0.476	
Case (%)		3162	871 (27.55)	1045 (33.05)	1246 (39.41)	24.26 (36.29)		
Non-case (%)		545,951	182,168 (33.37)	181,992 (33.33)	181,791 (33.30)	23.85 (35.28)		
		**Cruciferous vegetable intake**						
		Total	Low (0–7)	Medium (7–18)	High (> 18)	Mean [g/day (SD)]		
		549,113	183,045 (33.33)	183,031 (33.33)	183,037 (33.33)	26.69 (36.28)	< 0.001	
Case (%)		3162	939 (29.70)	1071 (33.87)	1152 (36.43)	33.95 (42.46)		
Non-case (%)		545,951	182,106 (33.36)	181,960 (33.33)	181,885 (33.32)	26.65 (36.24)		

^a^Calculated by chi-square (*χ*^2^) test for categorical variables and *t* test for continuous variables between bladder cancer cases and non-cases

^b^Age at the time of recruitment

^c^Pack-years was defined as the number of cigarettes smoked per day multiplying the years of smoking

*Abbreviations*: *SD*, standard deviation; *TNM*, tumour, nodes and metastasis classification; *g*, gram; *ml*, millilitre; *kcal*, kilocalorie; *MIBC*, muscle invasive bladder cancer; *NMIBC*, non-muscle invasive bladder cancer; *DDS*, dietary diversity score

*P* value < 0.05 was considered statistically significant

*P-*interaction < 0.05 was considered statistically significant

### Associations of vegetable intake with bladder cancer risk

#### Total vegetable intake and bladder cancer risk

For the total vegetable intake, no evidence of association was observed for the overall population and neither for the bladder cancer subtypes (overall: HR_model2; highest vs lowest tertile_ = 0.95, 95% CI = 0.85–1.07, *P* = 0.285 for trend, HR per 1 SD increment = 0.96, 95% CI = 0.91–1.01; NMIBC: HR_model2; highest vs lowest tertile_ = 1.01, 95% CI = 0.84–1.21, *P* = 0.404 for trend, HR per 1 SD increment = 0.97, 95% CI = 0.89–1.05; MIBC: HR_model2; highest vs lowest tertile_ = 0.99, 95% CI = 0.77–1.27, *P* = 0.582 for trend, HR per 1 SD increment = 0.97, 95% CI = 0.97–1.07) (Table 2). Sex-stratified analysis showed an inverse association between bladder cancer risk and high total vegetable intake among women (HR_model2; highest vs lowest tertile_ = 0.79, 95% CI = 0.64–0.98, *P* = 0.037 for trend; HR per 1 SD increment = 0.89, 95% CI = 0.81–0.99), while no association was observed among men (HR_model2; highest vs lowest tertile_ = 1.01, 95% CI = 0.88–1.14, *P* = 0.905 for trend; HR per 1 SD increment = 0.99, 95% CI = 0.94–1.03). Stratification for smoking only showed a marginally non-significant decreased bladder cancer risk per 1 SD increment of total vegetable intake among current smokers (HR_model2; highest vs lowest tertile_ = 0.95, 95% CI = 0.79–1.14, *P* = 0.051 for trend; HR per 1 SD increment = 0.90, 95% CI = 0.84–1.00). No such decline in risk could be observed among never or former smokers (respectively, HR_model2; highest vs lowest tertile_ = 1.05, 95% CI = 0.82–1.34, *P* = 0.787 for trend; HR per 1 SD increment = 1.00, 95% CI = 0.90–1.09), and former smokers (HR_model2; highest vs lowest tertile_ = 0.91, 95% CI = 0.77–1.09, *P* = 0.675 for trend; HR per 1 SD increment = 0.99, 95% CI = 0.94–1.09).

**Table 2:** Adjusted hazard ratios and 95% confidence intervals of bladder cancer according to intake of vegetable stratified by subtype, sex and smoking status

Study subgroup	Model adjustments	Vegetable intake (g/day)			HR per 1 SD increase (95% CI)	***P***-trend
		Low (0–117)	Medium (117–221)	High (> 221)		
**Overall**	No. Cases	962	916	1325		
	Model 1 ^d^	Ref.	1.02 (0.91–1.13)	0.96 (0.86–1.07)	0.97 (0.91–1.02)	0.417
	Model 2 ^d, e^	Ref.	1.01 (0.90–1.11)	0.95 (0.85–1.07)	0.96 (0.91–1.01)	0.285
**NMIBC**	No. Cases	392	390	485		
	Model 1 ^d^	Ref.	1.10 (0.94–1.28)	1.02 (0.85–1.22)	0.98 (0.90–1.06)	0.586
	Model 2 ^d, e^	Ref.	1.10 (0.94–1.28)	1.01 (0.84–1.21)	0.97 (0.89–1.05)	0.404
**MIBC**	No. Cases	167	213	393		
	Model 1 ^d^	Ref.	1.12 (0.89–1.41)	1.00 (0.78–1.28)	0.98 (0.89–1.08)	0.682
	Model 2 ^d, e^	Ref.	1.12 (0.89–1.41)	0.99 (0.77–1.27)	0.97 (0.88–1.07)	0.582
**Male**	No. Cases	717	646	1042		
	Model 1 ^d^	Ref.	1.03 (0.91–1.15)	1.02 (0.89–1.16)	1.00 (0.95–1.04)	0.826
	Model 2 ^d, e^	Ref.	1.02 (0.89–1.14)	1.01 (0.88–1.14)	0.99 (0.94–1.03)	0.905
**Female**	No. Cases	245	270	283		
	Model 1 ^d^	Ref.	0.89 (0.74–1.06)	0.79 (0.64–0.98)	0.89 (0.81–0.99)	0.034
	Model 2 ^d, e^	Ref.	0.89 (0.74–1.07)	0.79 (0.64–0.98)	0.89 (0.81–0.99)	0.037
**Never smoker**	No. Cases	191	187	271		
	Model 1 ^d^	Ref.	1.04 (0.85–1.28)	1.06 (0.84–1.35)	1.01 (0.91–1.10)	0.712
	Model 2 ^d, e^	Ref.	1.03 (0.83–1.26)	1.05 (0.82–1.34)	1.00 (0.90–1.09)	0.787
**Current smoker**	No. Cases	380	339	482		
	Model 1 ^d^	Ref.	1.00 (0.87–1.14)	0.96 (0.80–1.15)	0.91 (0.83–1.01)	0.073
	Model 2 ^d, e^	Ref.	0.99 (0.85–1.16)	0.95 (0.79–1.14)	0.90 (0.84–1.00)	0.051
**Former smoker**	No. Cases	410	402	554		
	Model 1 ^d^	Ref.	1.00 (0.86–1.16)	0.92 (0.78–1.10)	0.98 (0.95–1.07)	0.610
	Model 2 ^d, e^	Ref.	0.99 (0.85–1.15)	0.91 (0.77–1.09)	0.99 (0.94–1.09)	0.675

^d^Model 1: Adjusted for age (years, continuous), sex (male or female, if applicable), smoking (if applicable, smoking was defined as 0 (never smokers); 1 [current light smokers (i.e. smoking less than 20 pack-years)]; 2 [current heavy smokers (i.e. smoking more than 20 pack-years)]; 3 [current smokers (no information on pack-years)]; 4 [former light smokers (i.e. smokers who ceased smoking over 1 year prior and smoked less than 20 pack-years)]; 5 [former heavy smokers (i.e. smokers who ceased smoking over 1 year prior and smoked more than 20 pack-years)]; 6 [former smokers (smokers who ceased smoking over 1 year prior and no information on pack-years)]) and total energy intake (kcal/day, continuous)

^d, e^Model 2: Additionally, ethnicity (Caucasian or non-Caucasian, if applicable), alcohol intake (ml/day, continuous), fat intake (g/day, continuous), meat intake (g/day, continuous), sugar intake (g/day, continuous) and grain intake (g/day, continuous)

*Abbreviations*: *NMIBC*, non-muscle invasive bladder cancer; *MIBC*, muscle invasive bladder cancer; *SD*, standard deviation; *kcal*, kilocalorie; *g*, gram

Reference group was lowest intake (tertile 1)

*P*-trend < 0.05 was considered statistically significant

#### Subgroups of vegetable intake and bladder cancer risk

No evidence of association was found between the intake of non-starchy (HR_model2; highest vs lowest tertile_ = 0.95, 95% CI = 0.84–1.06, *P* = 0.356 for trend, HR per 1 SD increment = 0.98, 95% CI = 0.91–1.04), starchy (HR_model2; highest vs lowest tertile_ = 0.95, 95% CI = 0.82–1.09, *P* = 0.209 for trend, HR per 1 SD increment = 0.96, 95% CI = 0.89–1.02), green leafy (HR_model2; highest vs lowest tertile_ = 0.95, 95% CI = 0.84–1.07, *P* = 0.856 for trend, HR per 1 SD increment = 0.99, 95% CI = 0.96–1.03) or cruciferous vegetables (HR_model2; highest vs lowest tertile_ = 0.92, 95% CI = 0.81–1.03, *P* = 0.612 for trend, HR per 1 SD increment = 0.98, 95% CI = 0.93–1.00), and the risk of the bladder cancer in the overall population (Tables 3 and 4). Both bladder cancer subtype and smoking status stratified analyses showed similar results that no association for any of the vegetable subtype intake and bladder cancer risk could be observed. Among women, however, an inverse association with bladder cancer risk was observed for the intake of non-starchy vegetables (HR_model2; highest vs lowest tertile_ = 0.78, 95% CI = 0.63–0.97, *P* = 0.034 for trend; HR per 1 SD increment = 0.88, 95% CI = 0.79–0.98). No other vegetable subtype intake showed a significant association with bladder cancer risk neither in women nor in men.

**Table 3:** Adjusted hazard ratios and 95% confidence intervals of bladder cancer according to intake of non-starchy or starchy vegetable

Subgroup	Model	Non-starchy vegetable intake (g/day)			HR per 1 SD increase (95% CI)	***P***-trend	Starchy vegetable intake (g/day)			HR per 1 SD increase (95% CI)	***P***-trend
		Low (0–108)	Medium (108–211)	High (> 211)			Low (0–7)	Medium (7–18)	High (> 18)		
**Overall**	No. Cases	821	771	1570			692	491	331		
	Model 1 ^d^	Ref.	1.00 (0.91–1.10)	0.96 (0.85–1.08)	0.99 (0.95–1.06)	0.476	Ref.	0.94 (0.83–1.05)	0.94 (0.81–1.08)	0.95 (0.89–1.02)	0.153
	Model 2 ^d, e^	Ref.	0.99 (0.89–1.08)	0.95 (0.84–1.06)	0.98 (0.91–1.04)	0.356	Ref.	0.94 (0.84–1.06)	0.95 (0.82–1.09)	0.96 (0.89–1.02)	0.209
**NMIBC**	No. Cases	407	391	469			357	235	194		
	Model 1 ^d^	Ref.	1.05 (0.93–1.20)	0.97 (0.80–1.17)	0.99 (0.91–1.07)	0.722	Ref.	0.83 (0.70–0.98)	0.94 (0.77–1.13)	0.93 (0.85–1.01)	0.107
	Model 2 ^d, e^	Ref.	1.05 (0.92–1.20)	0.96 (0.79–1.16)	0.97 (0.89–1.06)	0.404	Ref.	0.83 (0.71–0.99)	0.95 (0.78–1.15)	0.93 (0.85–1.02)	0.131
**MIBC**	No. Cases	175	223	375			290	226	124		
	Model 1 ^d^	Ref.	1.03 (0.80–1.31)	0.99 (0.84–1.16)	0.98 (0.89–1.09)	0.713	Ref.	1.05 (0.88–1.25)	0.88 (0.70–1.11)	0.97 (0.87–1.08)	0.561
	Model 2 ^d, e^	Ref.	1.02 (0.80–1.31)	0.99 (0.84–1.16)	0.97 (0.88–1.08)	0.604	Ref.	1.05 (0.88–1.25)	0.89 (0.70–1.12)	0.98 (0.88–1.08)	0.639
**Male**	No. Cases	608	554	1212			571	406	245		
	Model 1 ^d^	Ref.	1.02 (0.90–1.14)	1.02 (0.91–1.18)	1.00 (0.94–1.06)	0.764	Ref.	0.96 (0.84–1.09)	0.93 (0.79–1.09)	0.96 (0.89–1.04)	0.307
	Model 2 ^d, e^	Ref.	1.02 (0.91–1.15)	1.01 (0.89–1.16)	0.99 (0.93–1.05)	0.979	Ref.	0.96 (0.84–1.09)	0.94 (0.80–1.11)	0.97 (0.89–1.04)	0.392
**Female**	No. Cases	213	217	358			121	85	86		
	Model 1 ^d^	Ref.	0.91 (0.75–1.07)	0.77 (0.62–0.97)	0.88 (0.80–0.99)	0.039	Ref.	0.85 (0.64–1.12)	0.95 (0.70–1.30)	0.93 (0.80–1.07)	0.288
	Model 2 ^d, e^	Ref.	0.90 (0.75–1.08)	0.78 (0.63–0.97)	0.88 (0.79–0.98)	0.034	Ref.	0.85 (0.64–1.12)	0.96 (0.71–1.31)	0.93 (0.81–1.07)	0.310
**Never smoker**	No. Cases	152	163	327			112	91	91		
	Model 1 ^d^	Ref.	1.06 (0.88–1.34)	1.07 (0.88–1.40)	1.01 (0.92–1.11)	0.619	Ref.	0.87 (0.66–1.15)	0.86 (0.64–1.17)	0.93 (0.82–1.07)	0.310
	Model 2 ^d, e^	Ref.	1.05 (0.88–1.35)	1.05 (0.87–1.38)	1.00 (0.91–1.10)	0.785	Ref.	0.87 (0.66–1.16)	0.86 (0.64–1.17)	0.90 (0.79–1.03)	0.116
**Current smoker**	No. Cases	371	309	494			270	149	67		
	Model 1 ^d^	Ref.	1.01 (0.87–1.18)	0.97 (0.80–1.16)	0.91 (0.85–1.02)	0.072	Ref.	0.93 (0.76–1.14)	0.98 (0.74–1.29)	0.97 (0.84–1.12)	0.691
	Model 2 ^d, e^	Ref.	1.00 (0.86–1.17)	0.95 (0.79–1.14)	0.90 (0.84–1.00)	0.058	Ref.	0.93 (0.76–1.14)	0.98 (0.74–1.30)	0.92 (0.79–1.07)	0.299
**Former smoker**	No. Cases	298	299	749			310	251	173		
	Model 1 ^d^	Ref.	0.94 (079–1.08)	0.91 (0.76–1.08)	0.99 (0.95–1.09)	0.604	Ref.	0.95 (0.81–1.13)	0.94 (0.77–1.15)	0.95 (0.87–1.04)	0.291
	Model 2 ^d, e^	Ref.	0.93 (0.78–1.09)	0.90 (0.75–1.05)	1.00 (0.94–1.11)	0.678	Ref.	0.96 (0.81–1.13)	0.95 (0.78–1.16)	0.96 (0.87–1.05)	0.357

^d^Model 1: Adjusted for age (years, continuous), sex (male or female, if applicable), smoking (if applicable, smoking was defined as: 0 (never smokers); 1 [current light smokers (i.e. smoking less than 20 pack-years)]; 2 [current heavy smokers (i.e. smoking more than 20 pack-years)]; 3 [current smokers (no information on pack-years)]; 4 [former light smokers (i.e. smokers who ceased smoking over 1 year prior and smoked less than 20 pack-years)]; 5 [former heavy smokers (i.e. smokers who ceased smoking over 1 year prior and smoked more than 20 pack-years)]; 6 [former smokers (smokers who ceased smoking over 1 year prior and no information on pack-years)]) and total energy intake (kcal/day, continuous)

^d, e^Model 2: Additionally, ethnicity (Caucasian or non-Caucasian, if applicable), alcohol intake (ml/day, continuous), fat intake (g/day, continuous), meat intake (g/day, continuous), sugar intake (g/day, continuous) and grain intake (g/day, continuous)

*Abbreviations*: *SD*, standard deviation; *ml*, millilitre; *kcal*, kilocalorie; *g*, gram

Reference group was lowest intake (tertile 1)

*P*-trend < 0.05 was considered statistically significant

**Table 4:** Adjusted hazard ratios and 95% confidence intervals of bladder cancer according to intake of green-leafy or cruciferous vegetable

Subgroup	Model	Green leaf vegetable intake (g/day)			HR per 1 SD increase (95% CI)	***P***-trend	Cruciferous vegetable intake (g/day)			HR per 1 SD increase (95% CI)	***P***-trend
		Low (0–5)	Medium (5–23)	High (> 23)			Low (0–7)	Medium (7–18)	High (> 18)		
**Overall**	No. Cases	871	1045	1246			939	1071	1152		
	Model 1 ^d^	Ref.	0.99 (0.89–1.07)	0.95 (0.85–1.08)	1.00 (0.95–1.05)	0.841	Ref.	0.97 (0.88–1.05)	0.93 (0.82–1.05)	1.00 (0.94–1.02)	0.579
	Model 2 ^d, e^	Ref.	0.98 (0.90–1.08)	0.95 (0.84–1.07)	0.99 (0.96–1.03)	0.856	Ref.	0.96 (0.85–1.04)	0.92 (0.81–1.03)	0.98 (0.93–1.00)	0.612
**NMIBC**	No. Cases	406	438	423			336	382	549		
	Model 1 ^d^	Ref.	0.99 (0.85–1.15)	0.95 (0.78–1.15)	0.98 (0.93–1.03)	0.465	Ref.	0.92 (0.78–1.07)	0.88 (0.72–1.07)	0.98 (0.90–1.06)	0.548
	Model 2 ^d, e^	Ref.	0.99 (0.85–1.15)	0.95 (0.78–1.16)	0.97 (0.89–1.06)	0.404	Ref.	0.91 (0.78–1.08)	0.95 (0.78–1.15)	0.93 (0.85–1.02)	0.131
**MIBC**	No. Cases	165	240	368			165	240	368		
	Model 1 ^d^	Ref.	0.92 (0.77–1.10)	0.82 (0.62–1.08)	0.98 (0.86–1.12)	0.771	Ref.	0.94 (0.71–1.25)	0.92 (0.71–1.19)	0.97 (0.89–1.06)	0.506
	Model 2 ^d, e^	Ref.	0.93 (0.77–1.11)	0.99 (0.61–1.07)	0.98 (0.86–1.12)	0.744	Ref.	0.95 (0.71–1.26)	0.93 (0.72–1.20)	0.97 (0.89–1.06)	0.479
**Male**	No. Cases	659	777	938			712	802	860		
	Model 1 ^d^	Ref.	1.00 (0.90–1.12)	0.95 (0.81–1.07)	0.98 (0.94–1.06)	0.136	Ref.	0.99 (0.86–1.09)	0.94 (0.79–1.08)	0.94 (0.88–1.07)	0.593
	Model 2 ^d, e^	Ref.	0.99 (0.89–1.11)	0.93 (0.80–1.06)	0.97 (0.93–1.05)	0.151	Ref.	0.98 (0.85–1.08)	0.92 (0.80–1.06)	0.93 (0.87–1.08)	0.624
**Female**	No. Cases	212	268	308			227	269	292		
	Model 1 ^d^	Ref.	1.01 (0.80–1.25)	1.01 (0.85–1.19)	0.91 (0.81–1.00)	0.064	Ref.	0.92 (0.71–1.15)	0.90 (0.74–1.11)	0.89 (0.80–1.00)	0.063
	Model 2 ^d, e^	Ref.	0.99 (0.79–1.23)	0.99 (0.82–1.18)	0.90 (0.79–1.00)	0.062	Ref.	0.91 (0.71–1.16)	0.89 (0.72–1.09)	0.91 (0.79–1.01)	0.059
**Never smoker**	No. Cases	180	213	249			179	229	234		
	Model 1 ^d^	Ref.	1.00 (0.81–1.24)	0.87 (0.67–1.13)	0.93 (0.82–1.04)	0.391	Ref.	1.02 (0.86–1.15)	1.01 (0.77–1.12)	0.92 (0.86–1.02)	0.167
	Model 2 ^d, e^	Ref.	0.99 (0.80–1.22)	0.85 (0.64–1.11)	0.92 (0.81–1.03)	0.401	Ref.	0.98 (0.76–1.10)	0.96 (0.74–1.09)	0.91 (0.80–1.01)	0.112
**Current smoker**	No. Cases	305	428	441			375	405	394		
	Model 1 ^d^	Ref.	1.06 (0.91–1.22)	0.97 (0.79–1.15)	0.95 (0.84–1.01)	0.382	Ref.	1.03 (0.89–1.21)	0.90 (0.72–1.08)	0.98 (0.91–1.15)	0.574
	Model 2 ^d, e^	Ref.	1.05 (0.90–1.20)	0.95 (0.77–1.14)	0.93 (0.83–1.02)	0.355	Ref.	1.03 (0.88–1.20)	0.89 (0.71–1.07)	0.98 (0.89–1.10)	0.512
**Former smoker**	No. Cases	386	404	556			385	437	524		
	Model 1 ^d^	Ref.	0.99 (0.80–1.17)	0.93 (0.80–1.08)	1.00 (0.94–1.11)	0.110	Ref.	0.95 (0.77–1.08)	0.89 (0.76–1.02)	0.98 (0.90–1.12)	0.472
	Model 2 ^d, e^	Ref.	0.97 (0.79–1.15)	0.92 (0.79–1.06)	0.99 (0.92–1.08)	0.109	Ref.	0.93 (0.80–1.07)	0.87 (0.75–1.01)	0.97 (0.87–1.11)	0.506

^d^Model 1: Adjusted for age (years, continuous), sex (male or female, if applicable), smoking (if applicable, smoking was defined as: 0 (never smokers); 1 [current light smokers (i.e. smoking less than 20 pack-years)]; 2 [current heavy smokers (i.e. smoking more than 20 pack-years)]; 3 [current smokers (no information on pack-years)]; 4 [former light smokers (i.e. smokers who ceased smoking over 1 year prior and smoked less than 20 pack-years)]; 5 [former heavy smokers (i.e. smokers who ceased smoking over 1 year prior and smoked more than 20 pack-years)]; 6 [former smokers (smokers who ceased smoking over 1 year prior and no information on pack-years)]) and total energy intake (kcal/day, continuous)

^d, e^Model 2: Additionally, ethnicity (Caucasian or non-Caucasian, if applicable), alcohol intake (ml/day, continuous), fat intake (g/day, continuous), meat intake (g/day, continuous), sugar intake (g/day, continuous) and grain intake (g/day, continuous)

*Abbreviations*: *SD*, standard deviation; *ml*, millilitre; *kcal*, kilocalorie; *g*, gram

Reference group was lowest intake (tertile 1)

*P*-trend < 0.05 was considered statistically significant

#### Individual vegetable type intake and bladder cancer risk

A significant decreased bladder cancer risk was observed for high intake of curly kale (HR_model2; highest vs lowest tertile_ = 0.80, 95% CI = 0.68–0.95; HR_model2; medium vs lowest tertile_ = 0.86, 95% CI = 0.73–1.00, *P* = 0.072 for trend; HR per 1 SD increment = 0.94, 95% CI = 0.87–1.01). However, this association disappeared after correction for multiple testing. No other individual vegetable type showed a significant association with bladder cancer risk (Additional file 1: Table S3).

#### Vegetable diversity intake and bladder cancer risk

The HRs and 95% CIs for different levels for the DDS and bladder cancer risk are presented in Table 5. There was no evidence of significant associations for DDS with bladder cancer risk (consuming 6–8 individual types versus 0–2 individual types: HR_model2_ = 0.95, 95% CI = 0.62–1.44, *P* = 0.340 for trend).

**Table 5:** Adjusted hazard ratios and 95% confidence intervals of bladder cancer according to diet diversity scores

Model adjustments	Diet diversity scores of vegetable				***P***-trend
	0–2	2–4	4–6	6–8	
No. Cases	27	962	1937	277	
Model 1 ^f^	Ref.	0.88 (0.60–1.32)	0.92 (0.61–1.38)	0.95 (0.62–1.45)	0.382
Model 2 ^f, g^	Ref.	0.87 (0.61–1.30)	0.92 (0.60–1.37)	0.95 (0.62–1.44)	0.340

^f^Model 1: Adjusted for age (years, continuous), sex (male or female, if applicable), smoking (if applicable, smoking was defined as: 0 (never smokers); 1 [current light smokers (i.e. smoking less than 20 pack-years)]; 2 [current heavy smokers (i.e. smoking more than 20 pack-years)]; 3 [current smokers (no information on pack-years)]; 4 [former light smokers (i.e. smokers who ceased smoking over 1 year prior and smoked less than 20 pack-years)]; 5 [former heavy smokers (i.e. smokers who ceased smoking over 1 year prior and smoked more than 20 pack-years)]; 6 [former smokers (smokers who ceased smoking over 1 year prior and no information on pack-years)]), total energy intake (kcal/day, continuous) and total vegetable intake

^f, g^Model 2: Additionally, ethnicity (Caucasian or non-Caucasian, if applicable), alcohol intake (ml/day, continuous), fat intake (g/day, continuous), meat intake (g/day, continuous), sugar intake (g/day, continuous) and grain intake (g/day, continuous)

*Abbreviations*: *NMIBC*, non-muscle invasive bladder cancer; *MIBC*, muscle invasive bladder cancer; *SD*, standard deviation; *kcal*, kilocalorie; *g*, gram

Reference group was lowest intake (tertile 1)

*P*-trend < 0.05 was considered statistically significant

#### Sensitivity analysis

Similar results for the intake of total vegetable with subgroups and bladder cancer risk was obtained from the sensitivity analysis when excluding bladder cancers diagnosed within the first 2 years after study entry (Additional file 1: Table S4). Stratified analyses by both sex and smoking status showed a similar inverse association between total and non-starchy vegetables and bladder cancer among never-smoking women (HR_model2; highest vs lowest tertile_ = 0.59, 95% CI = 0.39–0.90, *P*-trend = 0.013; HR_model2; highest vs lowest tertile_ = 0.65, 95% CI = 0.40–0.97, *P*-trend = 0.079, respectively) (Additional file 1: Table S5). In addition, the meta-analysis presented similar inverse association between total vegetable intake and bladder cancer for women (HR_model2; highest vs lowest tertile_ = 0.66, 95% CI = 0.49–0.83), while no association for overall population and men; in addition, only the EPIC study showed a reduced risk of bladder cancer in overall population (HR_model2; highest vs lowest tertile_ = 0.69, 95% CI = 0.61–0.79) and women (HR_model2; highest vs lowest tertile_ = 0.56, 95% CI = 0.45–0.71) (Additional file 1: Table S6 and Fig. S1).

## Discussion

In this large multi-centre prospective cohort study, we found an inverse association between intake of total and non-starchy vegetables and bladder cancer risk among women. This association, however, was not observed among men or for the overall population.

The observed null associations in the overall population and in men are in line with two previously conducted meta-analyses, including cohort studies only [26, 29], also showing no or little evidence of an inverse association of higher vegetable intake on bladder cancer risk. However, two other meta-analyses, including both case-control studies and cohort studies, showed an inverse association of vegetable intake and bladder cancer risk [27, 28]. A possible explanation for this observed discrepancy in results might be the inclusion of different study designs. Results obtained from long-term cohort studies, who prospectively obtained data, are suggested of substantially superior quality to those obtained from case-control studies. Another explanation could be that “total vegetable intake” may vary in its constituents (i.e. different individual vegetable types included) across different studies. However, our DDS analyses showed no association between the vegetable diversity and bladder cancer risk.

In the present study, a reduced risk of bladder cancer was found among women consuming high levels of “total vegetable” and non-starchy vegetables. Similar results were shown by other large prospective cohort studies, including EPIC, which also showed an inverse association of total vegetable intake among women only [22, 63, 64]. In addition, results from a previous BLEND study investigating the influence of vegetable intake on bladder cancer in case-control studies showed an inverse association among women, while a borderline decreased risk of bladder cancer was observed among men.

Although the evidence on the exact mechanism is lacking, some potential mechanisms could attribute to the observed difference between men and women: (1) hormones (e.g. oestrogen)—experimental studies showed that nutrients or chemicals obtained from vegetable have the potential to modulate the effect of endogenous hormones and thereby inhibit the development of bladder cancer among women [65]; (2) gene mutation—the protective effect of vegetables against cancer was found to be presented on some gene mutations only related to women [e.g. epidermal growth factor receptor (EGFR)] [66]; (3) urination habits—while men empty their bladder mainly in a standing position, women crouch over the toilet to empty the bladder without relaxation of the pelvic floor. This might cause urine retention, which results in a longer contact time between the metabolites of vegetable and the bladder [67]; and (4) behavioural vegetable intake—evidence showed that the vegetable intake in women is more persistently compared to men, which may lead to the consequence that the long-term benefit of vegetables is more pronounced in women [68–70]. However, it could not be excluded that residual confounding by other factors might explain the sex difference. Future research is, therefore, needed to clarify this finding and provide better understanding of how vegetable influence bladder cancer risk in terms of sexes.

The WCRF/AICR CUP reported suggestive but not conclusive protective effects of non-starchy vegetables on bladder cancer risk [71, 72]. In addition, by summarising 10 primary studies on non-starchy vegetable intake and bladder cancer risk, the WCRF/AICR CUP reported a borderline decreased bladder cancer risk per 1 serving/day increment of non-starchy vegetable intake (RR (relative risk) = 0.97, 95% CI = 0.94–1.00). The present study supports these findings by showing that a higher intake of non-starchy vegetables decreases the risk of bladder cancer among women. However, the biological mechanism for the role of non-starchy vegetables in the process of bladder carcinogenesis remains unclear.

In contrast to the inverse association of non-starchy vegetables on bladder cancer, a null association was observed for starchy vegetables. This finding is in line with a previous case-control study, which also reported a null association between higher starchy vegetables intake and bladder cancer risk. Starchy vegetables can raise blood sugar levels faster than non-starchy vegetables, resulting in an increased glycaemic loading and insulin response, and may thereby resist the suggested inverse association between vegetables and bladder cancer [73].

Green leafy vegetables contain several antioxidant nutrients, minerals, dietary fibre, phenols, flavonoids and phytochemicals [74–76], which may reduce oxidative stress and DNA damage caused by free radicals, and affect pathways controlling cell proliferation and apoptosis [77]. However, surprisingly no inverse association of this vegetable subtype could be observed in the present study or in any previously conducted prospective cohort studies [78, 79]. Null association in epidemiological studies may be the result of a limited amount range of intake, and thereby insufficient power to detect an association.

Cruciferous vegetables, in particular broccoli, contain high levels of glucosinolates. During food preparation, glucosinolates are transformed into isothiocyanates, which are suggested to favourably modify the carcinogen metabolism via inhibition of phase 1 enzymes and/or induction of phase 2 enzymes [80]. Several pooled results from case-control studies indeed showed an inverse association of cruciferous vegetables intake and bladder cancer risk [60, 81].

In the present study, however, this hypothesis could not be strengthened, since results showed a null association between cruciferous vegetable intake and bladder cancer risk. A similar null-finding was observed by the WCRF/AICR International’s CUP [19], who conducted a meta-analysis on results from 7 primary cohort studies, and by a meta-meta-analysis conducted by Al-Zalabani et al. [60] who pooled results from 2 meta-analyses of cohort studies. The discrepancy, between these epidemiological studies, might again be caused by the different study design of the included primary studies. Further studies with more optimal study design and dietary intake assessment methods (e.g. including more accurate assessments of individual vegetable types) need to be conducted in order clarify the associations between subtypes of vegetable and bladder cancer risk and the potential mechanisms.

The present study found a significant inverse association of high curly kale intake and bladder cancer risk. Although the exact mechanism of a protective effect of curly kale on carcinogenesis is lacking, it is reported to be one of the most nutrient-dense vegetables and might therefore help to prevent cancer [82]. It should be noted, however, that the observed association between curly kale and bladder cancer disappeared after correction for multiple testing. However, correction for multiple testing is likely too conservative when testing a priori hypotheses. Since we were able to formulate a plausible a priori hypothesis regarding the beneficial effect of curly kale [82], we believe that the observed protective effect is a true finding.

Null findings of other individual vegetable types might be caused by the fact that the intake of vegetable (sub) types individually does not reach a certain level which is needed to influence bladder cancer development. In addition, although BLEND provided enough statistical power for most individual vegetable subtypes, some subtypes (i.e. starchy vegetables) might have lacked sufficient power to detect small effect sizes. Future observational or experimental research aiming to investigate the effect of the intake of individual vegetable subtypes in preventing bladder cancer is warranted.

The effect of vegetables on bladder cancer risk may interact with smoking. Several experimental studies have reported a group of natural phytochemicals uniquely present in vegetables can modulate the smoking-related carcinogen metabolism and facilitate carcinogen detoxification, thus inhibiting smoking-related carcinogenesis [83–86]. This could potentially lead to a difference in the effect of vegetable intake on bladder cancer when stratifying for smoking status. Further research is needed to elucidate the potential interactive effect of smoking with vegetable intake on bladder cancer risk.

For the present study, data were pooled from 14 prospective cohorts, to obtain a sample size, large enough to permit detailed analyses with good precision. The study, however, also has some limitations: (a) other than age, sex and smoking, limited information was available on other possible risk factors, for the development of bladder cancer, such as body mass index (BMI), physical activity, socioeconomic status (SES), disinfection by-products, arsenic in the drinking water and occupational exposures to potentially carcinogenic chemicals; (b) some tumour subtype (i.e. muscle-invasive/non-muscle-invasive) information was missing, which hampered the statistical power required for stratified subgroup analyses; (c) people with a high intake of vegetables might have generally healthier lifestyles and diets than those with a low intake; thus, we could not rule out the possibility that some of the associations could be more likely due to a healthy lifestyle than to vegetable intake per se; (d) sample size for starchy and individual vegetables was smaller, which may have caused insufficient statistical power for detailed analyses; (e) although we found similar results after adjusting for potential dietary risk factors, it is still possible that the observed associations were confounded by other dietary constituents or additives associated with vegetable intake; (f) for most cohorts, the exposure variable was assessed by FFQs. Therefore, measurement error and misclassification of study participants in terms of the exposure and outcome are unavoidable. Moreover, since the level of detail in FFQs was varied in different cohorts, some individual vegetables investigated in this study could not be covered across all cohorts; (g) another concern is that competing risk could have influenced our results, and that assuming a higher vegetable intake would result in a lower risk of premature death could have biased our association towards null. However, due to lack of information on loss-to-follow up of non-cases (as well as cases), we were unable to perform competing risk analysis; (h) the present study sample consists mostly of Caucasians, and this may limit the generalizability of our results to other racial/ethnic populations or geographic regions; (i) although status as well as duration and intensity of smoking were taken into account in our analysis, the adjustment for smoking might still be imperfect due to differences in smoking practices (e.g. depth of inhalation or amount of inhalation), differences in types of smoke exposure, or lack of information on passive smoking [87]; (j) there is limited mechanistic evidence to interpret our findings; therefore, further studies are needed to clarify whether there is an underlying mechanism for the association between intake of vegetable and bladder cancer risk, especially for women; (k) another concern that needs to be considered is whether the results were caused by chance alone. Within this study, it is possible that the findings are a consequence of multiple testing. However, although large numbers of statistical techniques are available to correct for multiple comparison, it still remains a topic of discussion whether and how to correctly adjust for multiple testing [58, 59]. Given the fact that most of the observed significant findings (i.e. total vegetable and non-starchy vegetable) are supported by a plausible biological evidence, it is unlike these results are chance findings.

## Conclusion

In summary, the present study, including over 3200 cases of incident bladder cancer occurring in almost 0.6 million participants, indicates no overall relationship observed for the association of bladder cancer and intake of vegetables. Upon further sex-specific analyses, an association was observed among women. Future studies are needed to clarify the exact mechanism of this protective effect and should consider sex differences when investigating the association between vegetable intake and bladder cancer risk.

## Supplementary Information

**Additional file 1: Tables S1–6 & Fig. S1.** **Table S1.** Additional Baseline Characteristic of the Participant Cohort Studies. **Table S2.** The Categorisation of Subgroups of Vegetables in the Present Study. **Table S3.** Risk of Bladder Cancer According to Intake of Individual Types of Vegetable. **Table S4.** Risk of Bladder Cancer According to Intake of Total and Subgroup Vegetable (After Removing 350 Bladder Cancer Cases Diagnosed within 2 years). **Table S5.** Adjusted Hazard Ratios and 95% Confidence Intervals of Bladder Cancer According to Intake of Total and Non-starchy Vegetable Stratified by both Sex & Smoking Status based on Model 2. **Table S6.** Adjusted Hazard Ratios and 95% Confidence Intervals of Bladder Cancer According to Intake of Vegetable Stratified by Study Centre based on Model 2. **Figure S1.** Forest Plot of Meta-Analyses with HRs and 95% CIs for High vs. Low Intake of Total Vegetable with Bladder Cancer Risk A) Overall; B) Women; C) Men.

## Data Availability

The data that support the findings of this study are available on reasonable request pending approval from the corresponding author, AW. The data are not publicly available owing to their containing information that could compromise the privacy of research participants.

## Abbreviations

AIRC: American Institute for Cancer Research
BLEND: BLadder cancer Epidemiology and Nutritional Determinants
BMI: Body mass index
CI: Confidence interval
CUP: Continuous Update Project
DATS: Diallyl trisulfide
DDS: Diet diversity score
EPIC: European Prospective Investigation into Cancer and Nutrition cohort study
FFQ: Food frequency questionnaire
HR: Hazard ratio
ICD-O: International Classification of Diseases for Oncology
MCCS: Melbourne Collaborative Cohort Study
MIBC: Muscle-invasive bladder cancer
NLCS: NetherLands Cohort Study
NMIBC: Non-muscle-invasive bladder cancer
RERF-LSS: Radiation, Effects Research Foundation-Life Span Study
SAMC: S-Allylmercaptocysteine
SD: Standard deviation
SES: Socioeconomic status
VITAL: VITamins And Lifestyle cohort study
WCRF: World Cancer Research Fund
