# Correction: Vegetable intake and the risk of bladder cancer in the BLadder Cancer Epidemiology and Nutritional Determinants (BLEND) international study

**DOI:** 10.1186/s12916-026-04878-w

**Published:** 2026-04-22

**Authors:** Evan Yi-Wen Yu, Anke Wesselius, Siamak Mehrkanoon, Mieke Goosens, Maree Brinkman, Piet van den Brandt, Eric J. Grant, Emily White, Elisabete Weiderpass, Florence Le Calvez-Kelm, Marc J. Gunter, Inge Huybrechts, Elio Riboli, Anne Tjonneland, Giovanna Masala, Graham G. Giles, Roger L. Milne, Maurice P. Zeegers

**Affiliations:** 1 Department of Complex Genetics and Epidemiology, School of Nutrition and Translational Research in Metabolism, Maastricht University, Universiteitssingel 40 (Room C5.570), 6229 ER, Maastricht, the Netherlands; 2 CAPHRI School for Public Health and Primary Care, Maastricht University, Maastricht, The Netherlands; 3 Department of Data Science and Knowledge Engineering, Maastricht University, Maastricht, The Netherlands; 4 Department of General Practice, Katholieke Universiteit Leuven, ACHG-KU Leuven, Louvain, Belgium; 5 Department of Clinical Studies and Nutritional Epidemiology, Nutrition Biomed Research Institute, Melbourne, Australia; 6 Cancer Epidemiology Division, Cancer Council Victoria, 615 St Kilda Road, Melbourne, VIC 3004 Australia; 7 Department of Epidemiology, Schools for Oncology and Developmental Biology and Public Health and Primary Care, Maastricht University Medical Centre, Maastricht, The Netherlands; 8 Department of Epidemiology Radiation Effects Research Foundation, Hiroshima, Japan; 9 Fred Hutchinson Cancer Research Center, Seattle, WA USA; 10 International Agency for Research on Cancer World Health Organization, Lyon, France; 11 Department of Epidemiology and Biostatistics, School of Public Health, Imperial College London, London, UK; 12 Danish Cancer Society Research Center, Copenhagen, Denmark; 13 Department of Public Health, University of Copenhagen, Copenhagen, Denmark; 14 Molecular and Lifestyle Epidemiology Branch, Cancer Risk Factors and Lifestyle Epidemiology Unit, Institute for Cancer Research, Prevention and Clinical Network ISPRO, Florence, Italy; 15 Centre for Epidemiology and Biostatistics, Melbourne School of Population and Global Health, The University of Melbourne, 207 Bouverie Street, Melbourne, VIC 3010 Australia; 16 Precision Medicine, School of Clinical Sciences at Monash Health, Monash University, Clayton, VIC 3168 Australia; 17 School of Cancer Sciences, University of Birmingham, Birmingham, UK; 18 Department of Epidemiology & Biostatistics, School of Public Health, Southeast University, Nanjing, China

**Correction: BMC Med 19, 56 (2021)**

**https://doi.org/10.1186/s12916-021-01931-8**

The original article [1], did not include an additional institution affiliated with author Evan Yi-Wen Yu.

This additional affiliation should be noted as ‘Department of Epidemiology & Biostatistics, School of Public Health, Southeast University, Nanjing, China.’
