# Supplementary material for: Vegetable intake and the risk of bladder cancer in the BLadder Cancer Epidemiology and Nutritional Determinants (BLEND) international study
Source: BMC Med. 2021 Mar 9;19:56. doi: 10.1186/s12916-021-01931-8 (PMC7942172; doi:10.1186/s12916-021-01931-8)
Supplement: Supplementary file 1 — Additional file 1: Tables S1–6 & Fig. S1. Table S1. Additional Baseline Characteristic of the Participant Cohort Studies. Table S2. The Categorisation of Subgroups of Vegetables in the Present Study. Table S3. Risk of Bladder Cancer According to Intake of Individual Types of Vegetable. Table S4. Risk of Bladder Cancer According to Intake of Total and Subgroup Vegetable (After Removing 350 Bladder Cancer Cases Diagnosed within 2 years). Table S5. Adjusted Hazard Ratios and 95% Confidence Intervals of Bladder Cancer According to Intake of Total and Non-starchy Vegetable Stratified by both Sex & Smoking Status based on Model 2. Table S6. Adjusted Hazard Ratios and 95% Confidence Intervals of Bladder Cancer According to Intake of Vegetable Stratified by Study Centre based on Model 2. Figure S1. Forest Plot of Meta-Analyses with HRs and 95% CIs for High vs. Low Intake of Total Vegetable with Bladder Cancer Risk A) Overall; B) Women; C) Men. [file 12916_2021_1931_MOESM1_ESM.docx]

**Additionall file to “Vegetable Intake and the Risk of Bladder Cancer in the Bladder Cancer Epidemiology and Nutritional Determinants (BLEND) International Study”**

Evan Yi-Wen Yu, Anke Wesselius^Ψ^, Siamak Mehrkanoon, Mieke Goosens, Maree Brinkman, Piet van den Brandt, Eric J. Grant, Emily White, Elisabete Weiderpass, Florence Le Calvez-Kelm, Marc Gunter, Inge Huybrechts, Elio Riboli, Anne Tjonneland, Giovanna Masala, Graham G. Giles, Roger L. Milne, Maurice P. Zeegers

**^Ψ^ Corresponding Author:** Anke Wesselius, E-mail address:[anke.wesselius@maastrichtuniversity.nl](mailto:anke.wesselius@maastrichtuniversity.nl)

**Additional file 1: Table. S1** Additional Baseline Characteristic of the Participant Cohort Studies

**Additional file 1: Table. S2** The Categorization of Subgroups of Vegetables in the Present Study

**Additional file 1: Table. S3** Risk of Bladder Cancer According to Intake of Individual Types of Vegetable

**Additional file 1: Table. S4** Risk of Bladder Cancer According to Intake of Total and Subgroup Vegetable (After Removing 350 Bladder Cancer Cases Diagnosed within 2 years)

**Additional file 1: Table. S5** Adjusted Hazard Ratios and 95% Confidence Intervals of Bladder Cancer According to Intake of Total and Non-starchy Vegetable Stratified by both Sex & Smoking Status based on Model 2

**Additional file 1: Table. S6** Adjusted Hazard Ratios and 95% Confidence Intervals of Bladder Cancer According to Intake of Vegetable Stratified by Study Centre based on Model 2

**Additional file 1: Fig. S1** Forest Plot of Meta-Analyses with HRs and 95% CIs for High vs. Low Intake of Total Vegetable with Bladder Cancer Risk A) Overall; B) Women; C) Men

**Additional file 1: Table. S1** Additional Baseline Characteristic of the Participant Cohort Studies

| **Cohort** | **No. of Participants (%) ^a^** | **Follow-up Years ^b^** | **Age at Baseline ^c^** | **Person years** | **No. of Incidences Bladder Cancer (%)** | **Years to Diagnosis ^d^** | **% of Ever Smokers** | **Initiate year of baseline assessment** |
| --- | --- | --- | --- | --- | --- | --- | --- | --- |
| **EPIC (Europe)** |  |  |  |  |  |  |  |  |
| Denmark | 55,999 | 10.93 | 50-66 | 612,071 | 391 | 6.82 | 0.65 | 1993 |
| France | 64,837 | 10.39 | 42-71 | 673,560 | 31 | 7.25 | 0.29 | 1993 |
| Germany | 49,456 | 9.88 | 19-70 | 488,764 | 207 | 6.23 | 0.54 | 1994 |
| Italy | 45,201 | 11.23 | 24-78 | 507,735 | 186 | 6.89 | 0.55 | 1992 |
| Spain | 40,763 | 12.06 | 29-70 | 491,704 | 152 | 14.7 | 0.45 | 1992 |
| Sweden | 49,306 | 13.12 | 29-74 | 646,887 | 302 | 8.23 | 0.151 | 1991 |
| Netherlands | 37,080 | 11.81 | 20-70 | 438,039 | 107 | 7.36 | 0.62 | 1993 |
| United Kingdom | 75,014 | 11.14 | 20-98 | 835,339 | 248 | 7.05 | 0.44 | 1993 |
| Norway | 33,835 | 9.73 | 41-56 | 329,236 | 24 | 5.81 | 0.64 | 1998 |
| **NLCS (Netherlands)** | 5,245 | 14.06 | 55-70 | 73,756 | 877 | 9.34 | 0.68 | 1986 |
| **VITAL (USA)** | 69,290 | 6.75 | 50-76 | 467,413 | 346 | 3.87 | 0.52 | 2000 |
| **MCCS (Australia)** | 23,087 | 22.86 | 35-76 | 527,794 | 291 | 14.74 | 0.40 | 1990 |
| **RERF-LSS (Japan)** | 6,572 | 28.04 | 19-91 | 184,262 | 41 | 22.10 | 0.46 | 1960 |
| **Overall** | 555,685 | 11.22 | 19-98 | 6,276,560 | 3,253 | 8.24 | 0.50 | 1960-2000 |

Abbreviations: EPIC, European Prospective Investigation into Cancer; NLCS, The Netherlands Cohort Study; VITAL, VITamins And Lifestyle study; MCCS, Melbourne Collaborative Cohort Study; Radiation Effects Research Foundation-Life Span Study.

^a^ As a result of exclusion criteria, the cohort sizes and number of cases included in the pooled analyses may differ from original study-specific publications.

^b^ Median years of the time interval from two years after study entry to the date of the last follow-up (*e.g.* date of death, lost to follow-up, or study exit, whichever came first).

^c^ Age ranges at baseline (minimum to maximum).

^d^ Time-to-bladder cancer defined by median years of study entry to the date of diagnosis among bladder cancer cases.

**Additional file 1: Table. S2** The Categorization of Subgroups of Vegetables in the Present Study

| **Vegetable Subgroups** | **No. of Participants** | **No. of Cases (%)** | **Contained Individual Vegetables** |
| --- | --- | --- | --- |
| Starchy vegetable | 97,622 | 1,514 (1.56) | Cucurbita squash, pea, sweet corn, sweet potato, brassicas, tuber, beetroot, broad bean |
| Non-starchy vegetable | 549,113 | 3,162 (0.58) | asparagus, Brussels sprouts, broccoli, Chinese cabbage, cabbage, cauliflower, celery, cucumber, garlic, onion, pepper, spinach, tomato, courgette, lettuce, rhubarb, leek, carrot, aubergine, cucumber, pod and seed vegetable, French bean, okra, avocado, olive, edible fungi |
| Green-leafy vegetable | 577,543 | 3,162 (0.55) | rhubarb, spinach, brassicas, lettuce, curly kale, spinach, Chinese cabbage, cabbage, beetroot, pea |
| Cruciferous vegetable | 577,543 | 3,162 (0.55) | Tomato, lettuce, cauliflower, broccoli, Chinese cabbage, cabbage, cauliflower, curly kale, brassicas, Cucurbita squash |

**Additional file 1: Table. S3** Risk of Bladder Cancer According to Intake of Individual Types of Vegetable

| **Subgroup** | **Included Studies** | **Intake Tertiles** | **No. Case** | **Model 1 ^e^** | | |  |  | **Model 2 ^e, f^** | | |  |  |
| --- | --- | --- | --- | --- | --- | --- | --- | --- | --- | --- | --- | --- | --- |
|  |  |  |  | **HR (95% CI)** | **HR Per 1 SD Increase (95% CI)** | **P-trend** | **P-value** | **P-FDR ^g^** | **HR (95% CI)** | **HR Per 1 SD Increase (95% CI)** | **P-trend** | **P-value** | **P-FDR ^g^** |
| **Lettuce (g/day)** | NLCS, MCCS | Low (0-6) | 544 | Ref. | 1.07 (0.98-1.16) | 0.138 |  |  | Ref. | 1.08 (0.99-1.18) | 0.075 |  |  |
|  |  | Medium (6-16) | 418 | 1.03 (0.90-1.17) |  |  | 0.643 | 0.752 | 1.04 (0.91-1.18) |  |  | 0.646 | 0.741 |
|  |  | High (>16) | 206 | 1.11 (0.94-1.31) |  |  | 0.220 | 0.698 | 1.13 (0.96-1.34) |  |  | 0.182 | 0.740 |
| **Spinach (g/day)** | NLCS | Low (0-5) | 294 | Ref. | 1.01 (0.95-1.08) | 0.678 |  |  | Ref. | 1.01 (0.95-1.08) | 0.700 |  |  |
|  |  | Medium (5-12) | 287 | 0.97 (0.82-1.14) |  |  | 0.703 | 0.752 | 0.96 (0.81-1.13) |  |  | 0.754 | 0.814 |
|  |  | High (>12) | 296 | 1.02 (0.87-1.20) |  |  | 0.762 | 0.797 | 1.01 (0.86-1.19) |  |  | 0.969 | 0.983 |
| **Brassicas (g/day)** | NLCS, VITAL, EPIC, MCCS | Low (0-7) | 939 | Ref. | 0.98 (0.93-1.02) | 0.374 |  |  | Ref. | 0.97 (0.93-1.02) | 0.384 |  |  |
|  |  | Medium (7-25) | 1,071 | 0.94 (0.86-1.04) |  |  | 0.179 | 0.698 | 0.93 (0.84-1.02) |  |  | 0.357 | 0.740 |
|  |  | High (>25) | 1,152 | 0.90 (0.81-1.01) |  |  | 0.068 | 0.661 | 0.89 (0.80-1.00) |  |  | 0.049 | 0.555 |
| **Cauliflower (g/day)** | NLCS, MCCS | Low (0-2) | 202 | Ref. | 0.97 (0.92-1.02) | 0.237 |  |  | Ref. | 0.97 (0.92-1.02) | 0.259 |  |  |
|  |  | Medium (2-7) | 267 | 1.05 (0.87-1.26) |  |  | 0.629 | 0.752 | 1.05 (0.87-1.26) |  |  | 0.528 | 0.740 |
|  |  | High (>7) | 699 | 0.93 (0.78-1.11) |  |  | 0.374 | 0.716 | 0.93 (0.79-1.11) |  |  | 0.654 | 0.741 |
| **Broccoli (g/day)** | VITAL, MCCS | Low (0-3) | 228 | Ref. | 0.97 (0.89-1.05) | 0.435 |  |  | Ref. | 0.97 (0.90-1.06) | 0.535 |  |  |
|  |  | Medium (3-9) | 221 | 1.08 (0.89-1.30) |  |  | 0.532 | 0.739 | 1.09 (0.90-1.31) |  |  | 0.376 | 0.740 |
|  |  | High (>17) | 188 | 0.92 (0.75-1.12) |  |  | 0.418 | 0.716 | 0.93 (0.76-1.14) |  |  | 0.501 | 0.740 |
| **Chinese Cabbage (g/day)** | VITAL | Low (0) | 137 | Ref. | 0.96 (0.86-1.08) | 0.504 |  |  | Ref. | 0.97 (0.87-1.09) | 0.647 |  |  |
|  |  | Medium (0-2) | 99 | 1.03 (0.80-1.34) |  |  | 0.207 | 0.698 | 1.04 (0.80-1.35) |  |  | 0.787 | 0.836 |
|  |  | High (>2) | 110 | 0.91 (0.70-1.17) |  |  | 0.213 | 0.698 | 0.92 (0.71-1.20) |  |  | 0.594 | 0.740 |
| **Cabbage (g/day)** | NLCA, EPIC | Low (0-7) | 1,181 | Ref. | 1.00 (0.93-1.06) | 0.945 |  |  | Ref. | 1.01 (0.94-1.07) | 0.976 |  |  |
|  |  | Medium (7-26) | 864 | 0.99 (0.90-1.07) |  |  | 0.314 | 0.698 | 0.98 (0.90-1.08) |  |  | 0.415 | 0.740 |
|  |  | High (>26) | 480 | 1.02 (0.91-1.19) |  |  | 0.228 | 0.698 | 1.01 (0.91-1.19) |  |  | 0.327 | 0.740 |
| **Brussels Sprouts (g/day)** | NLCS, MCCS | Low (0-4) | 372 | Ref. | 0.96 (0.88-1.04) | 0.309 |  |  | Ref. | 0.96 (0.89-1.04) | 0.345 |  |  |
|  |  | Medium (4-10) | 447 | 1.01 (0.88-1.16) |  |  | 0.548 | 0.739 | 1.01 (0.88-1.16) |  |  | 0.946 | 0.975 |
|  |  | High (>10) | 349 | 0.93 (0.81-1.08) |  |  | 0.911 | 0.925 | 0.94 (0.81-1.09) |  |  | 0.351 | 0.740 |
| **Curly Kale (g/day)** | NLCS | Low (0-2) | 334 | Ref. | 0.94 (0.87-1.01) | 0.070 |  |  | Ref. | 0.94 (0.87-1.01) | 0.072 |  |  |
|  |  | Medium (2-4) | 264 | **0.86 (0.73-1.00)** |  |  | **0.025** | 0.340 | **0.86 (0.73-1.00)** |  |  | 0.021 | 0.357 |
|  |  | High (>4) | 279 | **0.81 (0.69-0.95)** |  |  | **0.003** | 0.204 | **0.80 (0.68-0.95)** |  |  | 0.002 | 0.102 |
| **Rhubarb (g/day)** | NLCS | Low (0) | 538 | Ref. | 0.97 (0.90-1.04) | 0.434 |  |  | Ref. | 0.97 (0.91-1.05) | 0.457 |  |  |
|  |  | Medium (0-2) | 118 | 0.99 (0.81-1.21) |  |  | 0.554 | 0.739 | 0.99 (0.81-1.22) |  |  | 0.983 | 0.983 |
|  |  | High (>2) | 221 | 0.96 (0.82-1.13) |  |  | 0.137 | 0.698 | 0.97 (0.83-1.14) |  |  | 0.628 | 0.740 |
| **Celery (g/day)** | MCCS | Low (0-1) | 100 | Ref. | 1.11 (1.00-1.23) | 0.048 |  |  | Ref. | 1.12 (1.01-1.24) | 0.032 |  |  |
|  |  | Medium (1-4) | 91 | 1.03 (0.77-1.37) |  |  | 0.674 | 0.752 | 1.04 (0.78-1.38) |  |  | 0.462 | 0.740 |
|  |  | High (>4) | 100 | 1.16 (0.88-1.53) |  |  | 0.583 | 0.752 | 1.18 (0.89-1.57) |  |  | 0.377 | 0.740 |
| **Asparagus (g/day)** | MCCS | Low (0-0.5) | 114 | Ref. | 1.06 (0.96-1.17) | 0.254 |  |  | Ref. | 1.07 (0.97-1.18) | 0.198 |  |  |
|  |  | Medium (0.5-2) | 81 | 0.91 (0.68-1.21) |  |  | 0.206 | 0.698 | 0.91 (0.68-1.21) |  |  | 0.207 | 0.740 |
|  |  | High (>2) | 96 | 1.08 (0.82-1.42) |  |  | 0.318 | 0.698 | 1.10 (0.83-1.45) |  |  | 0.320 | 0.740 |
| **Garlic (g/day)** | MCCS | Low (0-0.5) | 107 | Ref. | 0.97 (0.86-1.10) | 0.611 |  |  | Ref. | 0.97 (0.86-1.10) | 0.642 |  |  |
|  |  | Medium (0.5-1) | 107 | 1.22 (0.93-1.60) |  |  | 0.841 | 0.866 | 1.21 (0.92-1.59) |  |  | 0.709 | 0.778 |
|  |  | High (>1) | 77 | 0.98 (0.73-1.33) |  |  | 0.105 | 0.698 | 0.98 (0.72-1.34) |  |  | 0.211 | 0.740 |
| **Onion (g/day)** | NLCS | Low (0-11) | 442 | Ref. | 0.96 (0.90-1.02) | 0.211 |  |  | Ref. | 0.96 (0.89-1.03) | 0.212 |  |  |
|  |  | Medium (11-22) | 213 | 0.97 (0.86-1.10) |  |  | 0.488 | 0.739 | 1.03 (0.87-1.21) |  |  | 0.602 | 0.740 |
|  |  | High (>22) | 222 | 1.08 (1.06-1.09) |  |  | 0.013 | 0.221 | 0.92 (0.78-1.08) |  |  | 0.207 | 0.740 |
| **Leek (g/day)** | NLCS | Low (0-2) | 283 | Ref. | 1.05 (0.99-1.11) | 0.120 |  |  | Ref. | 1.05 (0.99-1.12) | 0.093 |  |  |
|  |  | Medium (2-11) | 300 | 1.14 (0.97-1.34) |  |  | 0.708 | 0.752 | 1.14 (0.97-1.35) |  |  | 0.613 | 0.740 |
|  |  | High (>11) | 294 | 1.12 (0.95-1.32) |  |  | 0.318 | 0.698 | 1.13 (0.96-1.33) |  |  | 0.296 | 0.740 |
| **Tuber (g/day)** | NLCS, MCCS, EPIC | Low (0) | 283 | Ref. | 0.99 (0.97-1.00) | 0.674 |  |  | Ref. | 0.99 (0.99-1.01) | 0.701 |  |  |
|  |  | Medium (0-40) | 300 | 0.90 (0.63-1.31) |  |  | 0.514 | 0.739 | 0.89 (0.62-1.25) |  |  | 0.601 | 0.740 |
|  |  | High (>40) | 294 | 0.89 (0.62-1.27) |  |  | 0.476 | 0.739 | 0.88 (0.60-1.23) |  |  | 0.489 | 0.740 |
| **Sweet potato (g/day)** | MCCS | Low (0-6) | 138 | Ref. | 1.07 (0.97-1.18) | 0.193 |  |  | Ref. | 1.08 (0.97-1.19) | 0.160 |  |  |
|  |  | Medium (6-16) | 65 | 0.76 (0.57-1.03) |  |  | 0.412 | 0.716 | 0.77 (0.57-1.03) |  |  | 0.368 | 0.740 |
|  |  | High (>16) | 88 | 1.12 (0.85-1.47) |  |  | 0.371 | 0.716 | 1.14 (0.86-1.50) |  |  | 0.402 | 0.740 |
| **Carrot (g/day)** | NLCS, TIVAL, MCCS | Low (0) | 570 | Ref. | 0.97 (0.90-1.04) | 0.389 |  |  | Ref. | 0.98 (0.91-1.06) | 0.602 |  |  |
|  |  | Medium (0-3) | 571 | 0.93 (0.83-1.04) |  |  | 0.624 | 0.752 | 0.94 (0.84-1.06) |  |  | 0.571 | 0.740 |
|  |  | High (>3) | 373 | 0.96 (0.84-1.10) |  |  | 0.249 | 0.698 | 0.98 (0.84-1.12) |  |  | 0.301 | 0.740 |
| **Beetroot (g/day)** | NLCS, MCCS | Low (0-0.5) | 355 | Ref. | 0.98 (0.94-1.02) | 0.322 |  |  | Ref. | 0.98 (0.94-1.02) | 0.358 |  |  |
|  |  | Medium (0.5-2.5) | 148 | 0.82 (0.67-0.99) |  |  | 0.042 | 0.476 | 0.82 (0.67-1.00) |  |  | 0.046 | 0.555 |
|  |  | High (>2.5) | 665 | 0.97 (0.85-1.11) |  |  | 0.633 | 0.752 | 0.98 (0.85-1.12) |  |  | 0.570 | 0.740 |
| **Tomato (g/day)** | NLCS, VITAL, MCCS | Low (0-18) | 649 | Ref. | 1.01 (0.94-1.07) | 0.852 |  |  | Ref. | 1.01 (0.95-1.08) | 0.651 |  |  |
|  |  | Medium (18-50) | 538 | 1.03 (0.92-1.16) |  |  | 0.686 | 0.752 | 1.04 (0.92-1.17) |  |  | 0.573 | 0.740 |
|  |  | High (>50) | 327 | 1.01 (0.88-1.17) |  |  | 0.978 | 0.978 | 1.03 (0.90-1.19) |  |  | 0.812 | 0.849 |
| **Aubergine (g/day)** | MCCS | Low (0) | 138 | Ref. | 1.03 (0.94-1.13) | 0.528 |  |  | Ref. | 1.03 (0.94-1.13) | 0.479 |  |  |
|  |  | Medium (0-1.5) | 48 | 0.98 (0.70-1.36) |  |  | 0.674 | 0.752 | 0.98 (0.70-1.37) |  |  | 0.682 | 0.760 |
|  |  | High (>1.5) | 105 | 1.32 (1.01-1.70) |  |  | 0.533 | 0.739 | 1.34 (1.03-1.75) |  |  | 0.491 | 0.740 |
| **Pepper (g/day)** | NLCS | Low (0) | 430 | Ref. | 1.00 (0.93-1.07) | 0.893 |  |  | Ref. | 1.00 (0.93-1.07) | 0.998 |  |  |
|  |  | Medium (0-3) | 214 | 1.08 (0.92-1.28) |  |  | 0.432 | 0.716 | 1.08 (0.92-1.27) |  |  | 0.411 | 0.740 |
|  |  | High (>3) | 233 | 1.02 (0.86-1.19) |  |  | 0.371 | 0.716 | 1.02 (0.87-1.20) |  |  | 0.298 | 0.740 |
| **Cucumber (g/day)** | MCCS | Low (0-3) | 93 | Ref. | 1.06 (0.96-1.18) | 0.233 |  |  | Ref. | 1.07 (0.97-1.19) | 0.165 |  |  |
|  |  | Medium (3-12) | 107 | 1.45 (1.09-1.91) |  |  | 0.684 | 0.752 | 1.46 (1.11-1.94) |  |  | 0.631 | 0.740 |
|  |  | High (>12) | 91 | 1.42 (0.86-1.19) |  |  | 0.593 | 0.752 | 1.41 (0.90-1.91) |  |  | 0.580 | 0.740 |
| **Courgette (g/day)** | MCCS | Low (0-1) | 106 | Ref. | 1.10 (0.99-1.21) | 0.078 |  |  | Ref. | 1.10 (1.00-1.22) | 0.061 |  |  |
|  |  | Medium (1-5) | 92 | 1.14 (0.86-1.51) |  |  | 0.247 | 0.698 | 1.14 (0.86-1.52) |  |  | 0.472 | 0.740 |
|  |  | High (>5) | 93 | 1.31 (0.99-1.75) |  |  | 0.312 | 0.698 | 1.35 (1.01-1.79) |  |  | 0.309 | 0.740 |
| **Cucurbita Squash (g/day)** | MCCS | Low (0-6) | 124 | Ref. | 0.92 (0.81-1.03) | 0.151 |  |  | Ref. | 0.91 (0.81-1.03) | 0.149 |  |  |
|  |  | Medium (6-19) | 84 | 0.83 (0.62-1.09) |  |  | 0.110 | 0.698 | 0.83 (0.63-1.10) |  |  | 0.212 | 0.740 |
|  |  | High (>19) | 83 | 0.81 (0.61-1.07) |  |  | 0.201 | 0.698 | 0.81 (0.60-1.07) |  |  | 0.179 | 0.740 |
| **Pod and Seed Vegetable (g/day)** | NLCS, VITAL, MCCS, EPIC | Low (0-0.2) | 836 | Ref. | 1.05 (1.00-1.10) | 0.069 |  |  | Ref. | 1.04 (0.99-1.10) | 0.073 |  |  |
|  |  | Medium (0.2-0.5) | 968 | 1.10 (0.99-1.21) |  |  | 0.273 | 0.698 | 1.11 (0.99-1.23) |  |  | 0.301 | 0.740 |
|  |  | High (>0.5) | 1,358 | 1.11 (0.99-1.25) |  |  | 0.196 | 0.698 | 1.12 (0.99-1.25) |  |  | 0.202 | 0.740 |
| **Pea (g/day)** | VITAL | Low (0-2) | 104 | Ref. | 1.00 (0.89-1.12) | 0.971 |  |  | Ref. | 1.01 (0.90-1.14) | 0.842 |  |  |
|  |  | Medium (2-7) | 119 | 1.03 (0.79-1.35) |  |  | 0.371 | 0.716 | 1.04 (0.80-1.35) |  |  | 0.323 | 0.740 |
|  |  | High (>7) | 123 | 1.16 (0.89-1.51) |  |  | 0.402 | 0.716 | 1.18 (0.90-1.54) |  |  | 0.411 | 0.740 |
| **Broad Bean (g/day)** | NLCS | Low (0) | 327 | Ref. | 1.06 (1.00-1.13) | 0.036 |  |  | Ref. | 1.06 (1.00-1.13) | 0.037 |  |  |
|  |  | Medium (0-5) | 232 | 1.23 (1.04-1.46) |  |  | 0.012 | 0.221 | 1.23 (1.04-1.46) |  |  | 0.007 | 0.159 |
|  |  | High (>5) | 318 | 1.24 (1.06-1.44) |  |  | 0.006 | 0.204 | 1.23 (1.05-1.44) |  |  | 0.003 | 0.102 |
| **French Bean (g/day)** | NLCS, VITAL | Low (0-5) | 216 | Ref. | 1.00 (0.94-1.06) | 0.957 |  |  | Ref. | 1.00 (0.94-1.06) | 0.984 |  |  |
|  |  | Medium (5-13) | 370 | 0.91 (0.77-1.09) |  |  | 0.109 | 0.698 | 0.91 (0.77-1.09) |  |  | 0.108 | 0.740 |
|  |  | High (>13) | 637 | 0.94 (0.79-1.11) |  |  | 0.211 | 0.698 | 0.93 (0.79-1.11) |  |  | 0.214 | 0.740 |
| **Sweet Corn (g/day)** | TIVAL, MCCS | Low (0-2) | 240 | Ref. | 1.01 (0.93-1.10) | 0.851 |  |  | Ref. | 1.01 (0.93-1.10) | 0.825 |  |  |
|  |  | Medium (2-7) | 219 | 1.17 (0.97-1.41) |  |  | 0.475 | 0.739 | 1.18 (0.98-1.43) |  |  | 0.469 | 0.740 |
|  |  | High (>7) | 178 | 1.10 (0.90-1.35) |  |  | 0.501 | 0.739 | 1.11 (0.90-1.36) |  |  | 0.512 | 0.740 |
| **Okra (g/day)** | VITAL | Low (0) | 143 | Ref. | 1.02 (0.91-1.15) | 0.698 |  |  | Ref. | 1.05 (0.93-1.17) | 0.447 |  |  |
|  |  | Medium (0-4) | 105 | 1.15 (0.89-1.48) |  |  | 0.607 | 0.752 | 1.17 (0.91-1.51) |  |  | 0.584 | 0.740 |
|  |  | High (>4) | 98 | 0.96 (0.74-1.25) |  |  | 0.423 | 0.716 | 1.01 (0.77-1.31) |  |  | 0.396 | 0.740 |
| **Avocado (g/day)** | VITAL, MCCS | Low (0) | 319 | Ref. | 1.00 (0.92-1.08) | 0.977 |  |  | Ref. | 1.01 (0.93-1.09) | 0.839 |  |  |
|  |  | Medium (0-2) | 141 | 1.01 (0.82-1.23) |  |  | 0.275 | 0.698 | 1.01 (0.83-1.24) |  |  | 0.293 | 0.740 |
|  |  | High (>2) | 177 | 1.04 (0.86-1.26) |  |  | 0.316 | 0.698 | 1.07 (0.88-1.29) |  |  | 0.274 | 0.740 |
| **Olive (g/day)** | MCCS | Low (0-0.4) | 96 | Ref. | 1.05 (0.95-1.15) | 0.335 |  |  | Ref. | 1.05 (0.95-1.15) | 0.364 |  |  |
|  |  | Medium (0.4-2) | 89 | 1.10 (0.82-1.48) |  |  | 0.412 | 0.716 | 1.11 (0.83-1.49) |  |  | 0.406 | 0.740 |
|  |  | High (>2) | 106 | 1.21 (0.91-1.61) |  |  | 0.507 | 0.739 | 1.22 (0.91-1.63) |  |  | 0.511 | 0.740 |
| **Edible Fungi (g/day)** | NLCS, MCCS, EPIC | Low (0-1) | 1,040 | Ref. | 0.99 (0.93-1.04) | 0.624 |  |  | Ref. | 0.99 (0.94-1.02) | 0.640 |  |  |
|  |  | Medium (1-5) | 1,030 | 1.02 (0.94-1.10) |  |  | 0.273 | 0.698 | 1.02 (0.93-1.12) |  |  | 0.254 | 0.740 |
|  |  | High (>5) | 742 | 1.01 (0.91-1.14) |  |  | 0.178 | 0.698 | 1.00 (0.90-1.11) |  |  | 0.183 | 0.740 |

^e^ Model 1: Adjusted for age (years, continuous), sex (male or female, if applicable), smoking (smoking was defined as: 0 (never smokers); 1 [current light smokers (i.e. smoking less than 20 pack-years)]; 2 [current heavy smokers (i.e. smoking more than 20 pack-years)]; 3 [current smokers (no information on pack-years)]; 4 [former light smokers (i.e. smokers who ceased smoking over 1 year prior and smoked less than 20 pack-years)]; 5 [former heavy smokers (i.e. smokers who ceased smoking over 1 year prior and smoked more than 20 pack-years)]; 6 [former smokers (smokers who ceased smoking over 1 year prior and no information on pack-years)]), and total energy intake (kcal/day, continuous).

^e, f^ Model 2: Additionally, ethnicity (Caucasian or non-Caucasian, if applicable), alcohol intake (ml/day, continuous), fat intake (g/day, continuous), meat intake (g/day, continuous), sugar intake (g/day, continuous), and grain intake (g/day, continuous).

^g^ P-FDR was obtained after multiple testing by using false discovery rate method.

Abbreviation: HR, hazard ratio; SD, standard deviation; CI, confidence interval; g, gram; kcal, kilocalorie; EPIC, European Prospective Investigation into Cancer; NLCS, The Netherlands Cohort Study; VITAL, VITamins And Lifestyle study; MCCS, Melbourne Collaborative Cohort Study; Radiation Effects Research Foundation-Life Span Study.

The intervals of tertiles were categorized as open lower values and ended upper values.

Reference group was lowest intake (tertile 1). P-trend <0.05 was considered statistically significant.

**Additional file 1: Table. S4** Risk of Bladder Cancer According to Intake of Total and Subgroup Vegetable (After Removing 350 Bladder Cancer Cases Diagnosed within 2 years)

| **Subgroup** | **Intake Tertiles** | **No. Case** | **Model 1 ^e^** | | | **Model 2 ^e, f^** | | |
| --- | --- | --- | --- | --- | --- | --- | --- | --- |
|  |  |  | **HR (95% CI)** | **HR Per 1 SD Increase (95% CI)** | **P-trend** | **HR (95% CI)** | **HR Per 1 SD Increase (95% CI)** | **P-trend** |
| **Total Vegetable (g/day)** | Low (0-177) | 832 | Ref. | 0.99 (0.93-1.03) | 0.326 | Ref. | 0.97 (0.92-1.02) | 0.233 |
|  | Medium (117-221) | 813 | 1.00 (0.90-1.10) |  |  | 1.00 (0.90-1.10) |  |  |
|  | High (>221) | 1,208 | 0.96 (0.85-1.08) |  |  | 0.81 (0.70-0.93) |  |  |
| **Total Vegetable for Women(g/day)** | Low (0-177) | 218 | Ref. | 0.88 (0.79-0.98) | 0.023 | Ref. | 0.88 (0.79-0.98) | 0.026 |
|  | Medium (117-221) | 243 | 0.88 (0.73-1.06) |  |  | 0.88 (0.73-1.07) |  |  |
|  | High (>221) | 264 | 0.78 (0.62-0.96) |  |  | 0.78 (0.62-0.97) |  |  |
| **Non-starchy Vegetable (g/day)** | Low (0-108) | 725 | Ref. | 0.98 (0.93-1.03) | 0.701 | Ref. | 0.97 (0.92-1.02) | 0.274 |
|  | Medium (108-211) | 683 | 0.99 (0.89-1.11) |  |  | 0.99 (0.89-1.11) |  |  |
|  | High (>211) | 1,404 | 0.97 (0.85-1.11) |  |  | 0.97 (0.85-1.11) |  |  |
| **Non-starchy Vegetable for Women (g/day)** | Low (0-108) | 192 | Ref. | 0.88 (0.79-0.98) | 0.027 | Ref. | 0.88 (0.79-0.99) | 0.026 |
|  | Medium (108-211) | 200 | 0.88 (0.73-1.07) |  |  | 0.89 (0.73-1.07) |  |  |
|  | High (>211) | 323 | 0.77 (0.61-0.96) |  |  | 0.77 (0.61-0.96) |  |  |
| **Starchy Vegetable (g/day)** | Low (0-7) | 619 | Ref. | 0.95 (0.89-1.02) | 0.202 | Ref. | 0.96 (0.89-1.03) | 0.248 |
|  | Medium (7-18) | 429 | 0.92 (0.81-1.04) |  |  | 0.93 (0.82-1.05) |  |  |
|  | High (>18) | 294 | 0.92 (0.79-1.07) |  |  | 0.93 (0.80-1.09) |  |  |
| **Green Leafy Vegetable (g/day)** | Low (0-5) | 740 | Ref. | 0.99 (0.93-1.04) | 0.648 | Ref. | 0.99 (0.93-1.04) | 0.631 |
|  | Medium (5-23) | 942 | 1.01 (0.91-1.11) |  |  | 1.01 (0.92-1.12) |  |  |
|  | High (>23) | 1,130 | 0.93 (0.82-1.06) |  |  | 0.93 (0.82-1.06) |  |  |
| **Cruciferous Vegetable (g/day)** | Low (0-8) | 815 | Ref. | 0.97 (0.93-1.02) | 0.278 | Ref. | 0.97 (0.93-1.02) | 0.262 |
|  | Medium (8-26) | 956 | 1.01 (0.91-1.11) |  |  | 0.96 (0.87-1.08) |  |  |
|  | High (>26) | 1,041 | 0.93 (0.82-1.06) |  |  | 0.93 (0.82-1.06) |  |  |

^e^ Model 1: Adjusted for age (years, continuous), sex (male or female, if applicable), smoking (smoking was defined as: 0 (never smokers); 1 [current light smokers (i.e. smoking less than 20 pack-years)]; 2 [current heavy smokers (i.e. smoking more than 20 pack-years)]; 3 [current smokers (no information on pack-years)]; 4 [former light smokers (i.e. smokers who ceased smoking over 1 year prior and smoked less than 20 pack-years)]; 5 [former heavy smokers (i.e. smokers who ceased smoking over 1 year prior and smoked more than 20 pack-years)]; 6 [former smokers (smokers who ceased smoking over 1 year prior and no information on pack-years)]), and total energy intake (kcal/day, continuous).

^e, f^ Model 2: Additionally, ethnicity (Caucasian or non-Caucasian, if applicable), alcohol intake (ml/day, continuous), fat intake (g/day, continuous), meat intake (g/day, continuous), sugar intake (g/day, continuous), and grain intake (g/day, continuous).

Abbreviation: HR, hazard ratio; SD, standard deviation; CI, confidence interval; g, gram; kcal, kilocalorie.

The intervals of tertiles were categorized as open lower values and ended upper values.

Reference group was lowest intake (tertile 1).

P-trend <0.05 were considered statistically significant.

**Additional file 1: Table. S5** Adjusted Hazard Ratios and 95% Confidence Intervals of Bladder Cancer According to Intake of Total and Non-starchy Vegetable Stratified by both Sex & Smoking Status based on Model 2

| **Study Subgroup** | **Model Adjustments** | **Vegetable Intake (g/day)** | | | **P-_trend_** |
| --- | --- | --- | --- | --- | --- |
|  |  | Low | Medium | High |  |
| **Total Vegetable** |  |  |  |  |  |
| **Male** |  |  |  |  |  |
| **Never Smoker** | No. Cases | 102 | 87 | 127 |  |
|  | Model 2 ^e, f^ | Ref. | 1.08 (0.79-1.46) | 1.06 (0.78-1.31) | 0.076 |
| **Current Smoker** | No. Cases | 285 | 251 | 423 |  |
|  | Model 2 ^e, f^ | Ref. | 0.97 (0.76-1.28) | 0.94 (0.78-1.9) | 0.501 |
| **Former Smoker** | No. Cases | 330 | 308 | 492 |  |
|  | Model 2 ^e, f^ | Ref. | 0.99 (0.75-1.23) | 0.93 (0.77-1.16) | 0.437 |
| **Female** |  |  |  |  |  |
| **Never Smoker** | No. Cases | 89 | 100 | 144 |  |
|  | Model 2 ^e, f^ | Ref. | 0.78 (0.56-1.08) | 0.59 (0.39-0.90) | 0.013 |
| **Current Smoker** | No. Cases | 95 | 88 | 59 |  |
|  | Model 2 ^e, f^ | Ref. | 0.96 (0.67-1.37) | 0.92 (0.66-1.29) | 0.840 |
| **Former Smoker** | No. Cases | 61 | 82 | 80 |  |
|  | Model 2 ^e, f^ | Ref. | 0.82 (0.60-1.12) | 0.58 (0.40-0.86) | 0.007 |
| **Non-starchy Vegetable** |  |  |  |  |  |
| **Male** |  | 73 | 82 | 161 |  |
| **Never Smoker** | No. Cases | Ref. | 1.23 (0.81-1.89) | 1.06 (0.73-1.53) | 0.328 |
|  | Model 2 ^e, f^ | 283 | 233 | 418 |  |
| **Current Smoker** | No. Cases | Ref. | 0.97 (0.79-1.14) | 0.81 (0.58-1.12) | 0.201 |
|  | Model 2 ^e, f^ | 252 | 239 | 633 |  |
| **Former Smoker** | No. Cases | Ref. | 1.12 (0.83-1.26) | 0.92 (0.78-1.10) | 0.029 |
|  | Model 2 ^e, f^ |  |  |  |  |
| **Female** |  |  |  |  |  |
| **Never Smoker** | No. Cases | 79 | 81 | 160 |  |
|  | Model 2 ^e, f^ | Ref. | 0.81 (0.57-1.14) | 0.65 (0.40-0.97) | 0.079 |
| **Current Smoker** | No. Cases | 88 | 76 | 82 |  |
|  | Model 2 ^e, f^ | Ref. | 1.08 (0.70-1.38) | 0.93 (0.64-1.35) | 0.329 |
| **Former Smoker** | No. Cases | 46 | 60 | 116 |  |
|  | Model 2 ^e, f^ | Ref. | 1.05 (0.76-1.26) | 0.91 (0.78-1.17) | 0.714 |

^e,f^ Model 2: Adjusted for age (years, continuous), sex (male or female, if applicable), smoking (smoking was defined as: 0 (never smokers); 1 [current light smokers (i.e. smoking less than 20 pack-years)]; 2 [current heavy smokers (i.e. smoking more than 20 pack-years)]; 3 [current smokers (no information on pack-years)]; 4 [former light smokers (i.e. smokers who ceased smoking over 1 year prior and smoked less than 20 pack-years)]; 5 [former heavy smokers (i.e. smokers who ceased smoking over 1 year prior and smoked more than 20 pack-years)]; 6 [former smokers (smokers who ceased smoking over 1 year prior and no information on pack-years)]), and total energy intake (kcal/day, continuous), ethnicity (Caucasian or non-Caucasian, if applicable) , alcohol intake (ml/day, continuous), fat intake (g/day, continuous), meat intake (g/day, continuous), sugar intake (g/day, continuous), and grain intake (g/day, continuous).

Abbreviation: HR, hazard ratio; SD, standard deviation; CI, confidence interval; g, gram; kcal, kilocalorie.

The intervals of tertiles were categorized as open lower values and ended upper values.

Reference group was lowest intake (tertile 1).

P-trend <0.05 was considered statistically significant.

**Additional file 1: Table. S6** Adjusted Hazard Ratios and 95% Confidence Intervals of Bladder Cancer According to Intake of Vegetable Stratified by Study Centre based on Model 2

| **Study Subgroup** | **Model Adjustments** | **Vegetable Intake (g/day)** | | | ***P*-_trend_** |
| --- | --- | --- | --- | --- | --- |
|  |  | Low | Medium | High |  |
| **NLCS** | No. Cases | 261 | 195 | 321 |  |
|  | Model 2 ^e, f^ | Ref. | 0.98 (0.83-1.16) | 0.93 (0.78-1.11) | 0.329 |
| **RERF-LSS** | No. Cases | 26 | 13 | 2 |  |
|  | Model 2 ^e, f^ | Ref. | 0.65 (0.33-1.29) | 0.97 (0.23-4.13) | 0.423 |
| **VITAL** | No. Cases | 121 | 124 | 101 |  |
|  | Model 2 ^e, f^ | Ref. | 1.09 (0.85-1.41) | 1.09 (0.82-1.46) | 0.389 |
| **MCCS** | No. Cases | 101 | 103 | 87 |  |
|  | Model 2 ^e, f^ | Ref. | 1.27 (0.96-1.69) | 1.11 (0.82-1.50) | 0.170 |
| **EPIC** | No. Cases | 585 | 608 | 455 |  |
|  | Model 2 ^e, f^ | Ref. | 0.90 (0.80-1.01) | 0.69 (0.61-0.79) | <0.001 |

^e,f^ Model 2: Adjusted for age (years, continuous), sex (male or female, if applicable), smoking (smoking was defined as: 0 (never smokers); 1 [current light smokers (i.e. smoking less than 20 pack-years)]; 2 [current heavy smokers (i.e. smoking more than 20 pack-years)]; 3 [current smokers (no information on pack-years)]; 4 [former light smokers (i.e. smokers who ceased smoking over 1 year prior and smoked less than 20 pack-years)]; 5 [former heavy smokers (i.e. smokers who ceased smoking over 1 year prior and smoked more than 20 pack-years)]; 6 [former smokers (smokers who ceased smoking over 1 year prior and no information on pack-years)]), and total energy intake (kcal/day, continuous), ethnicity (Caucasian or non-Caucasian, if applicable) , alcohol intake (ml/day, continuous), fat intake (g/day, continuous), meat intake (g/day, continuous), sugar intake (g/day, continuous), and grain intake (g/day, continuous).

Abbreviation: HR, hazard ratio; SD, standard deviation; CI, confidence interval; g, gram; kcal, kilocalorie.

The intervals of tertiles were categorized as open lower values and ended upper values.

Reference group was lowest intake (tertile 1).

P-trend <0.05 was considered statistically significant.


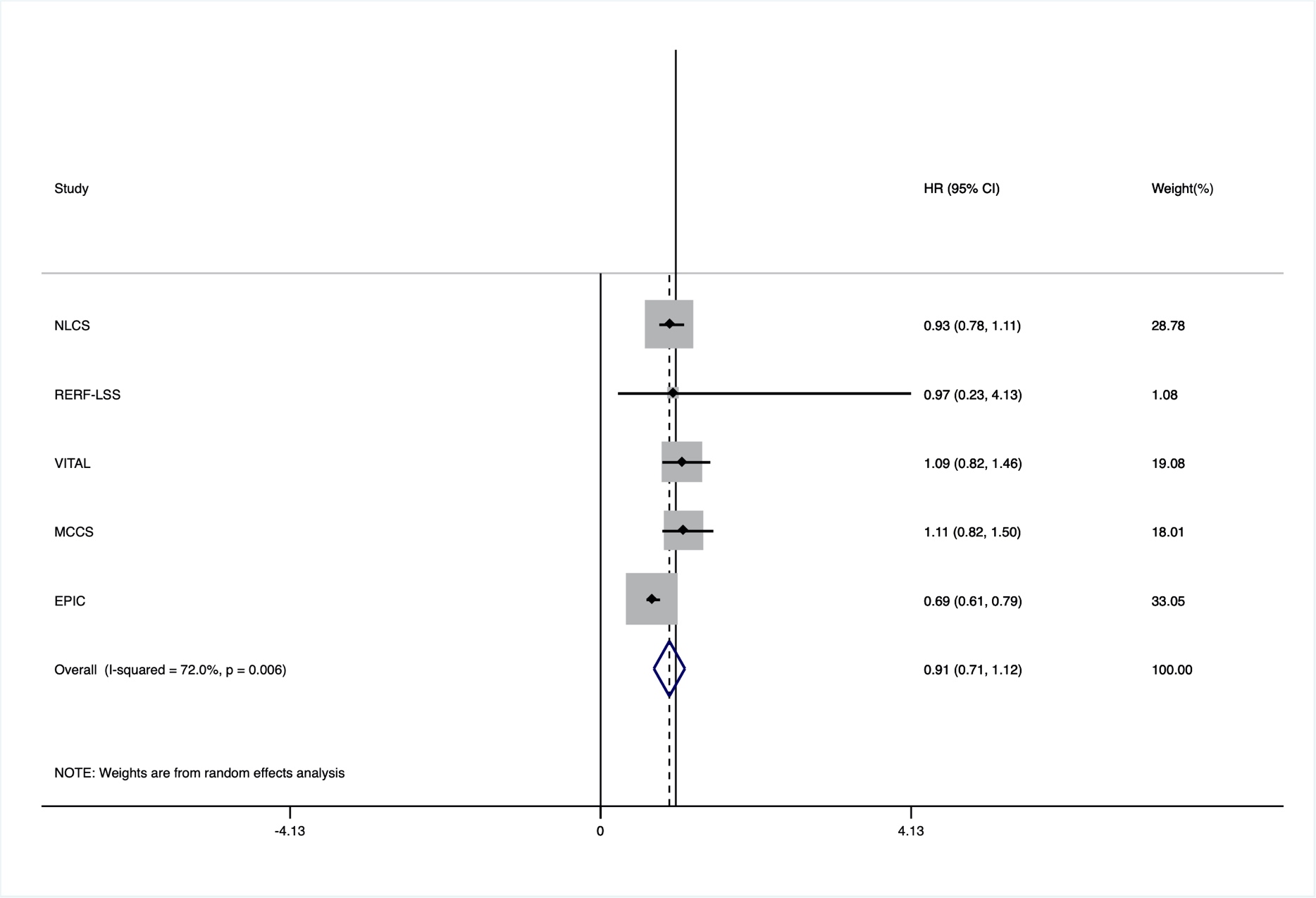


1. Overall

**
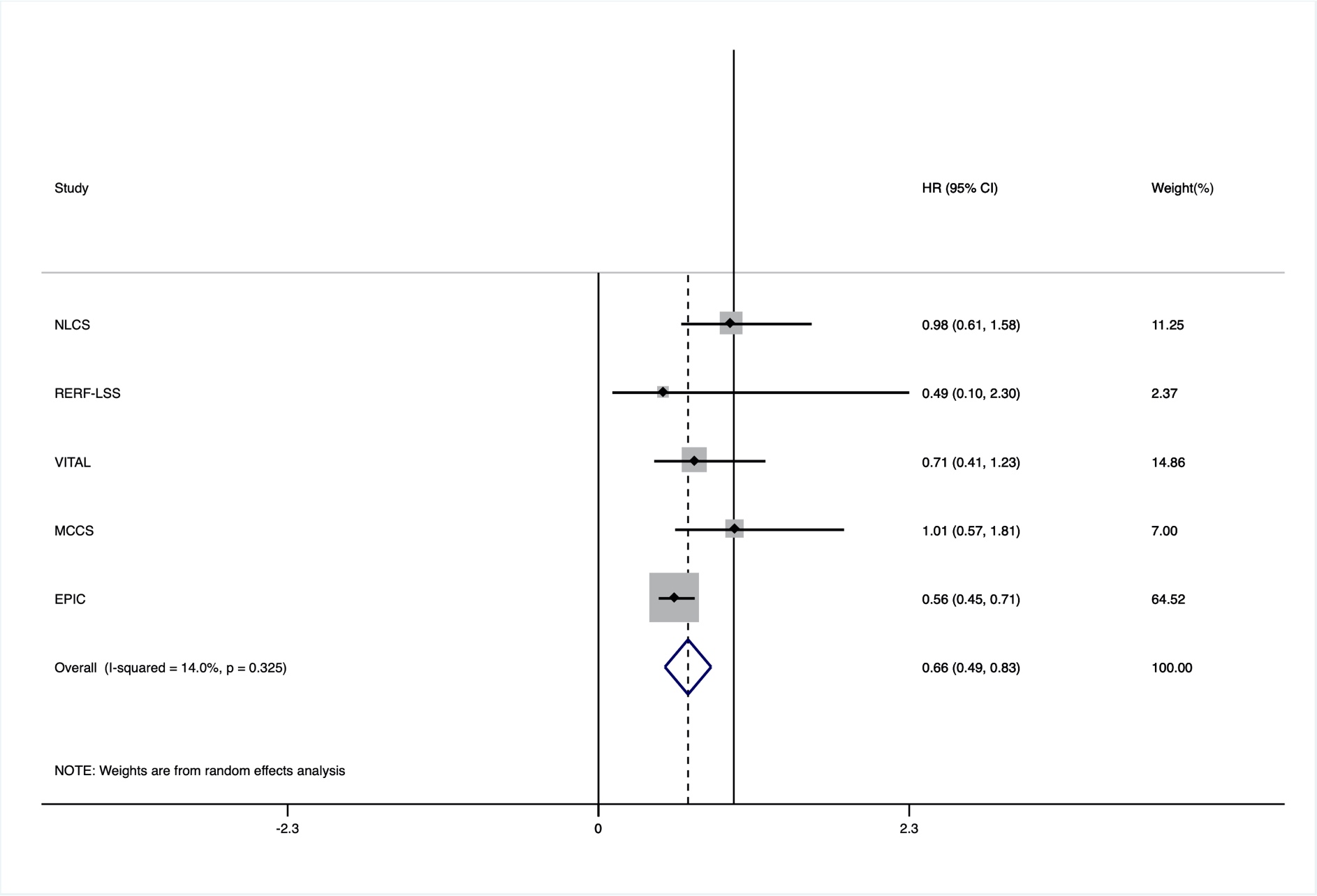
**

1. Women

**
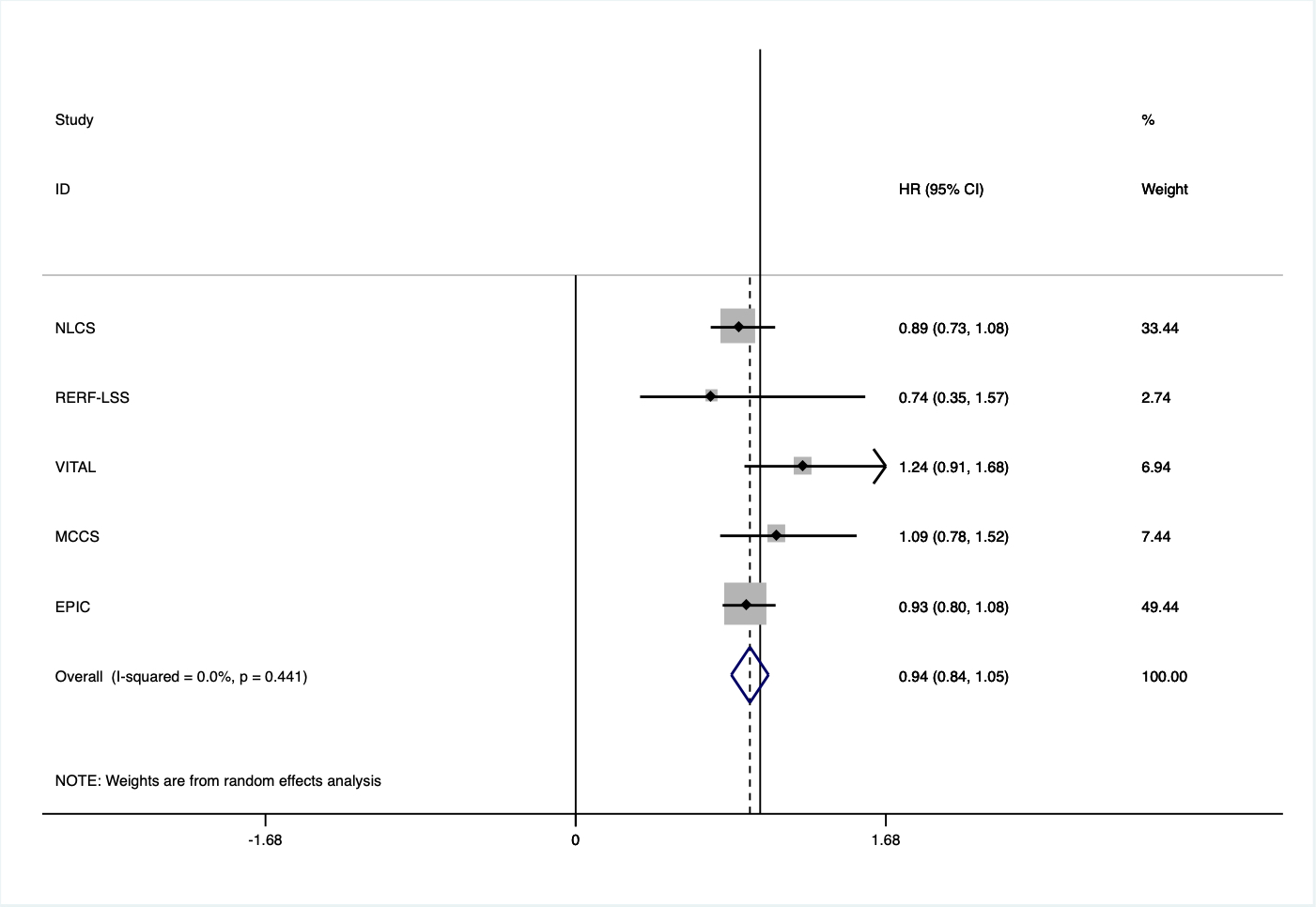
**

1. Men

**Additional file 1: Fig. S1** Forest Plot of Meta-Analyses with HRs and 95% CIs for High vs. Low Intake of Total Vegetable with Bladder Cancer Risk A) Overall; B) Women; C) Men

Diamond dots denote the hazard ratios (HRs); Horizontal lines represent the 95% confidence intervals (CIs); Weights (grey squares) are from random effects analyses.

Adjusted for age (years, continuous), sex (male or female, if applicable), smoking (smoking was defined as: 0 (never smokers); 1 [current light smokers (i.e. smoking less than 20 pack-years)]; 2 [current heavy smokers (i.e. smoking more than 20 pack-years)]; 3 [current smokers (no information on pack-years)]; 4 [former light smokers (i.e. smokers who ceased smoking over 1 year prior and smoked less than 20 pack-years)]; 5 [former heavy smokers (i.e. smokers who ceased smoking over 1 year prior and smoked more than 20 pack-years)]; 6 [former smokers (smokers who ceased smoking over 1 year prior and no information on pack-years)]), and total energy intake (kcal/day, continuous), ethnicity (Caucasian or non-Caucasian, if applicable) , alcohol intake (ml/day, continuous), fat intake (g/day, continuous), meat intake (g/day, continuous), sugar intake (g/day, continuous), and grain intake (g/day, continuous).

Abbreviation: HR, hazard ratio; CI, confidence interval; g, gram; kcal, kilocalorie; ml, millilitre.

Reference group was lowest intake (tertile 1).
